# Increased plasma XOR activity induced by NAFLD/NASH and its possible involvement in vascular neointimal proliferation

**DOI:** 10.1172/jci.insight.144762

**Published:** 2021-09-08

**Authors:** Yusuke Kawachi, Yuya Fujishima, Hitoshi Nishizawa, Takashi Nakamura, Seigo Akari, Takayo Murase, Takuro Saito, Yasuhiro Miyazaki, Hirofumi Nagao, Shiro Fukuda, Shunbun Kita, Naoto Katakami, Yuichiro Doki, Norikazu Maeda, Iichiro Shimomura

**Affiliations:** 1 Department of Metabolic Medicine, Graduate School of Medicine, Osaka University, Suita, Osaka, Japan.; 2 Sanwa Kagaku Kenkyusho Co., Ltd., Inabe, Mie, Japan.; 3 Department of Gastroenterological Surgery,; 4 Department of Adipose Management, and; 5 Department of Metabolism and Atherosclerosis, Graduate School of Medicine, Osaka University, Suita, Osaka, Japan.

**Keywords:** Hepatology, Metabolism, Atherosclerosis, Endothelial cells

## Abstract

Xanthine oxidoreductase (XOR) is an enzyme that catalyzes hypoxanthine to xanthine and xanthine to uric acid, respectively. However, the underlying mechanisms of increased plasma XOR and its pathological roles in systemic diseases, such as atherosclerosis, are not fully understood. In this study, we found that changes in plasma XOR activity after bariatric surgery closely associated with those in liver enzymes, but not with those in BMI. In a mouse model of nonalcoholic fatty liver disease/steatohepatitis (NAFLD/NASH), plasma XOR activity markedly increased. Besides, purine catabolism was accelerated in the plasma per se of NASH mice and human patients with high XOR activity. In our NASH mice, we observed an increased vascular neointima formation consisting of dedifferentiated vascular smooth muscle cells (SMCs), which was significantly attenuated by topiroxostat, a selective XOR inhibitor. In vitro, human liver S9–derived XOR promoted proliferation of SMCs with phenotypic modulation and induced ROS production by catabolizing hypoxanthine released from human endothelial cells. Collectively, the results from human and mouse models suggest that increased plasma XOR activity, mainly explained by excess hepatic leakage, was involved in the pathogenesis of vascular injury, especially in NAFLD/NASH conditions.

## Graphical abstract

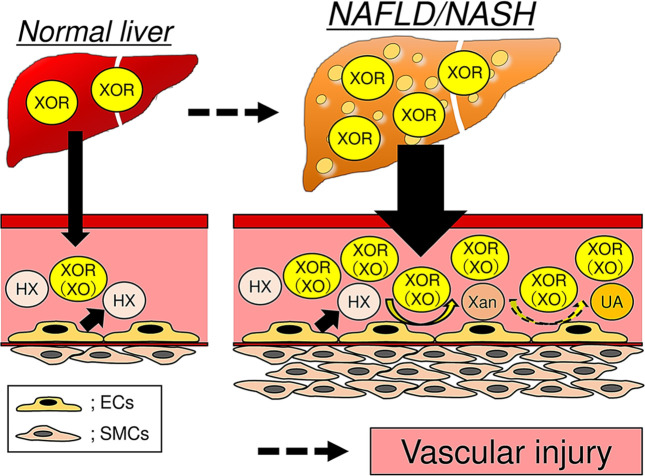

## Introduction

Accumulating evidence from epidemiological studies suggests that hyperuricemia, the etiology of gout, is associated with chronic kidney diseases (1, 2) and a potential risk factor for cardiovascular diseases (CVDs; refs. 3, 4). Patients with hyperuricemia consist of heterogeneous populations, such as the overproduction type in liver and reduced renal/extrarenal excretion type (5), the latter of which is often related to genetic abnormalities in transporters of uric acid (6). On the other hand, the overproduction type of hyperuricemia is closely associated with visceral fat–based metabolic syndrome, including type 2 diabetes mellitus, hypertension, dyslipidemia, and nonalcoholic fatty liver disease/steatohepatitis (NAFLD/NASH; ref. 7). Although significant reductions in serum uric acid have been achieved through the recent progress in urate-lowering therapy, such as xanthine oxidoreductase (XOR) inhibitors, the clinical effects of antihyperuricemic agents on CVDs remain controversial (8–10). As such, further studies are needed to identify the potential underlying mechanisms of these conflicting outcomes.

XOR is a rate-limiting enzyme that catalyzes conversions from hypoxanthine to xanthine and from xanthine to uric acid. Although the gene expression of murine XOR is found in various tissues, including adipose tissue, human XOR localizes mainly in liver and intestines (11). XOR is initially synthesized as the xanthine dehydrogenase (XDH) form, and then XDH is converted to xanthine oxidase (XO) under pathophysiological conditions, such as tissue hypoxia (12, 13), or after being released into the circulation (14, 15). Moreover, XO is known to generate ROS through the production of O^2–^ and H_2_O_2_. In vitro analysis demonstrated that XO binds to negatively charged glycosaminoglycans at the apical surface of vascular endothelial cells (ECs; refs. 16, 17); therefore, XO was hypothesized to be involved in vascular endothelial damage (18–20).

Recent methodological advances have made it possible to measure plasma XOR activity in humans (21), and serial studies described the association of plasma XOR with multiple clinical parameters, such as BMI, insulin resistance, hyperuricemia, and liver dysfunction (22, 23). We just recently reported that, across 2 weeks of hospitalized treatment, plasma XOR activity in diabetic patients was strongly and independently associated with liver transaminases (aspartate aminotransferase, AST; and alanine aminotransferase, ALT; ref. 24). However, it remains unclear which of these metabolic disorders has a causal relationship with increased plasma XOR activity and whether high plasma XOR is implicated in the pathogenesis of systemic diseases, such as atherosclerosis.

In this study, we demonstrate the close association of plasma XOR activity with liver enzymes in patients after bariatric surgery, increased XOR activity in plasma of NAFLD/NASH models, and its possible involvement in the development of vascular neointimal proliferation.

## Results

### Close associations between changes in plasma XOR activity and those in liver transaminases, but not in BMI, after bariatric surgery, in obese patients.

To clinically identify factors associated with plasma XOR activity, we conducted a follow-up study on morbidly obese patients who underwent bariatric surgery, an intervention by which marked alterations in systemic metabolism are expected. We examined changes in plasma XOR activity and their related clinical parameters short (1 week) and long (1 year) periods after surgery. The baseline characteristics of the patients (8 men/4 women) are shown in Supplemental Table 1 (supplemental material available online with this article; https://doi.org/10.1172/jci.insight.144762DS1). As shown in Supplemental Table 2, baseline plasma XOR activity was positively correlated with serum AST and ALT in the univariate analysis.

**Figure 1:**
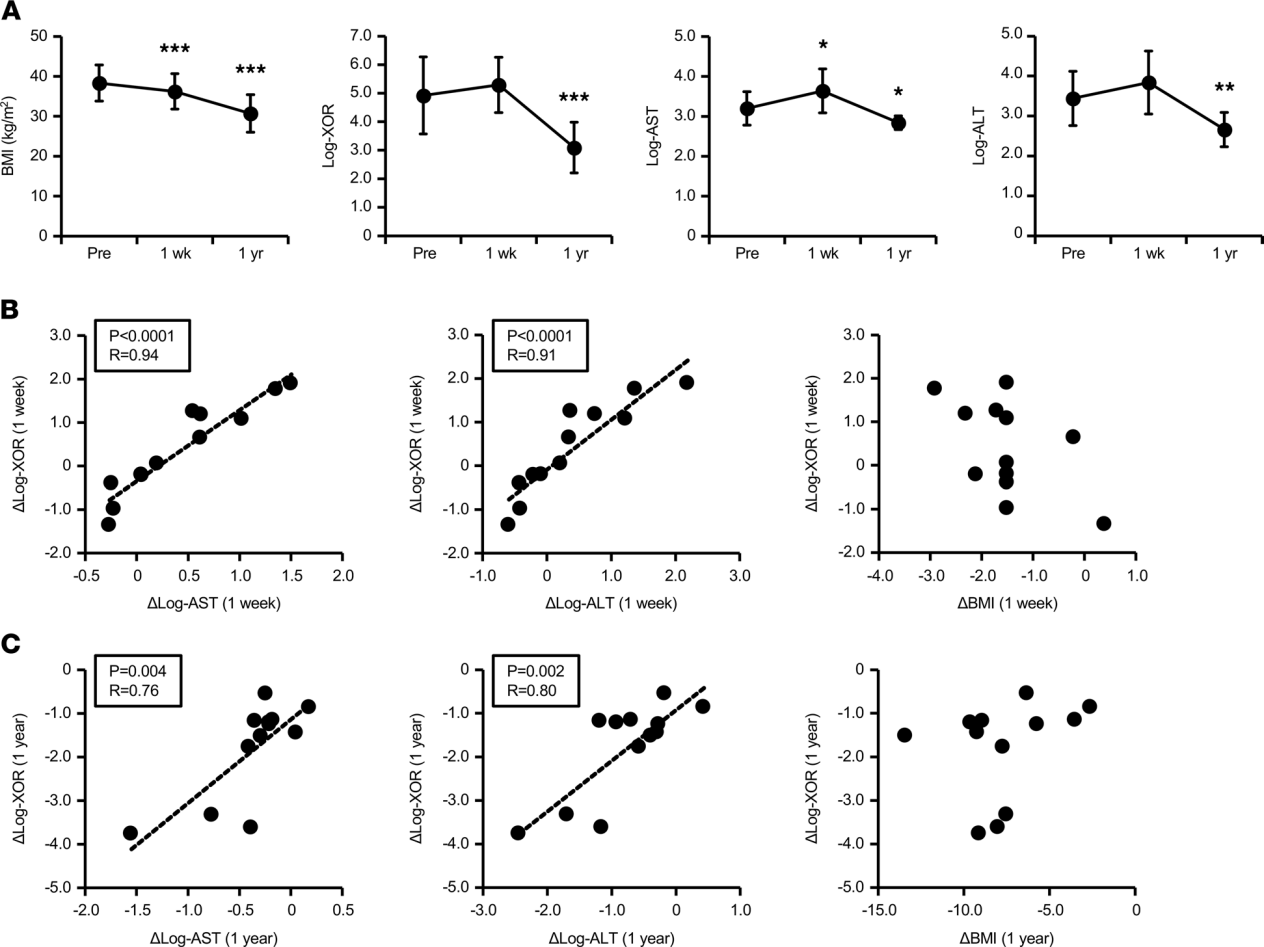
Significant reductions in plasma XOR activity after bariatric surgery and correlations with liver transaminases. Each clinical parameter was examined in 12 obese patients before (Pre) bariatric surgery and 1 week and 1 year after bariatric surgery. Plasma XOR activity was measured using the LC/TQMS method. (**A**) Serial changes in BMI, plasma XOR activity, serum AST, and ALT after the surgery. *P < 0.05, **P < 0.01, and ***P < 0.001 vs. Pre (paired *t* test). (**B** and **C**) Pearson’s correlation coefficients between changes in plasma XOR activity and those in serum AST, ALT, and BMI at 1 week (**B**) and 1 year (**C**) after the surgery. XOR, xanthine oxidoreductase; LC/TQMS, triple quadrupole mass spectrometry and liquid chromatography; AST, aspartate aminotransferase; ALT, alanine aminotransferase.****

Changes in clinical parameters from baseline to 1 week or 1 year after bariatric surgery are shown in Supplemental Table 3. As expected, BMI markedly decreased from 38.3 kg/m^2^ to 36.9 kg/m^2^ 1 week (*P* = 0.0003) and to 30.7 kg/m^2^ 1 year (*P* < 0.0001) after the surgery (Figure 1A), accompanied by significant improvements in multiple metabolic parameters. One year after the surgery, we observed significant reductions in plasma XOR activity (*P* = 0.0001; Figure 1A). Both serum AST and ALT also significantly decreased after 1 year (Figure 1A), similar to XOR activity. Subsequent correlation analysis (Supplemental Table 4) revealed that only changes in AST and ALT, but not in BMI, had significant positive associations with those in plasma XOR activity both 1 week (Figure 1B) and 1 year (Figure 1C) after the surgery. Of note, no significant correlation was observed between the changes in XOR activity and those in plasma uric acid levels (Supplemental Table 4). These clinical observations suggest that increased plasma XOR activity was predominantly associated with liver dysfunction, such as NAFLD/NASH, rather than obesity per se.

### A marked increase in plasma XOR activity in NAFLD/NASH mice without obesity.

**Figure 2:**
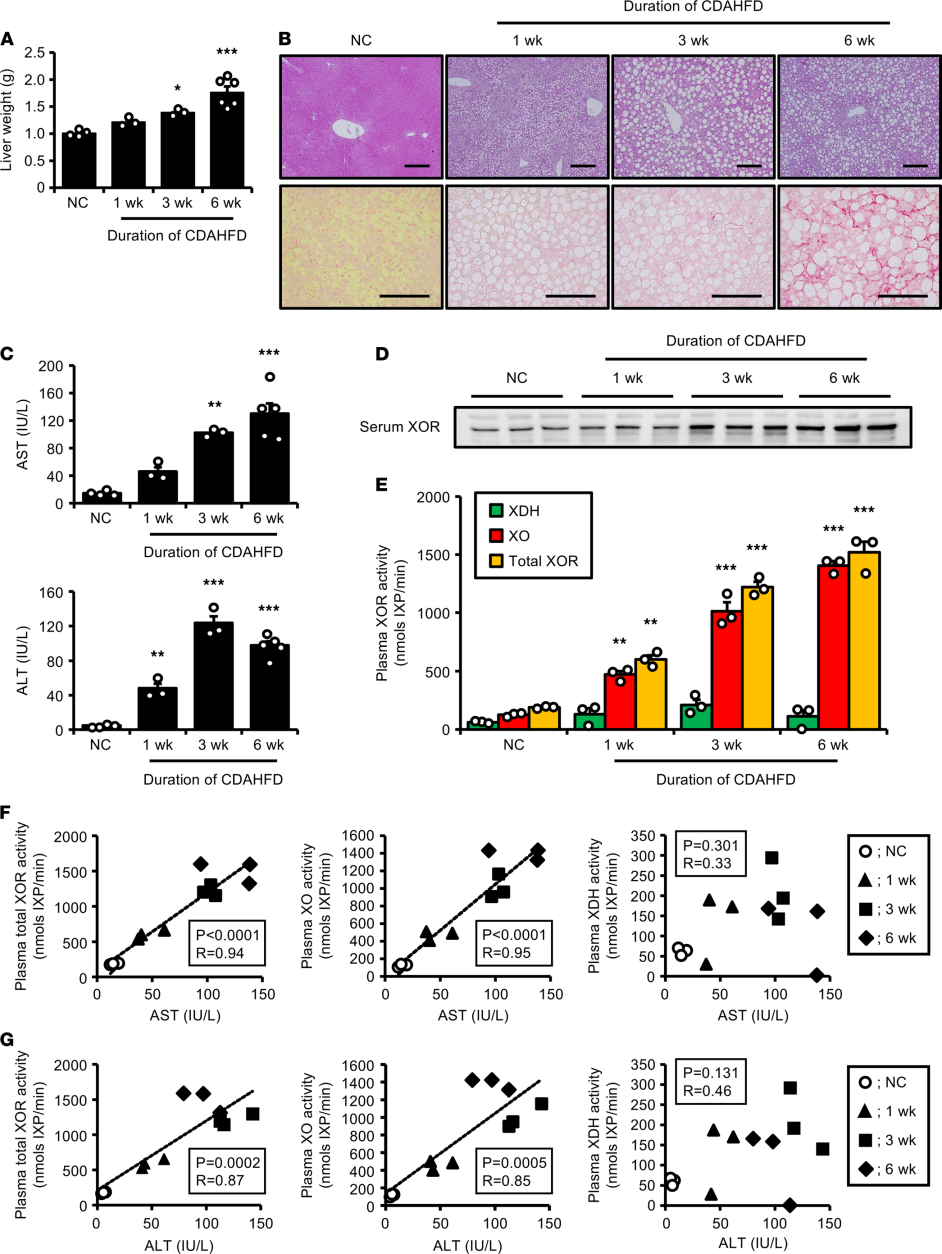
Increased plasma XOR activity in NAFLD/NASH mice fed CDAHFD. Male C57BL/6J mice were fed a CDAHFD for up to 6 weeks from 8 weeks of age. (**A**) Liver weight gain after CDAHFD feeding. *n* = 4; NC for 6 weeks, *n* = 3; CDAHFD for 1 week and 3 weeks, and *n* = 6; CDAHFD for 6 weeks. (**B**) Representative liver sections stained by H&E (upper panels) and Sirius red (lower panels). Scale bars: 100 μm. (**C**) Serum AST and ALT after CDAHFD feeding. *n* = 4; NC for 6 weeks, *n* = 3; CDAHFD for 1 week and 3 weeks, and *n* = 5; CDAHFD for 6 weeks. (**D**) Immunoblots for serum XOR protein. (**E**) Plasma XDH, XO, and total XOR activity after CDAHFD feeding. The HPLC-FLD method was used to distinguish between XO activity and total XOR (XO plus XDH) activity. XDH activity was calculated by subtracting XO activity from total XOR activity. *n* = 3 for each group. Data are shown as mean ± SEM. *P < 0.05, **P < 0.01, and ***P < 0.001 vs. NC (1-way ANOVA with Dunnett’s post hoc test). (**F** and **G**) Pearson’s correlation coefficient was used to examine the correlation between serum AST (**F**) or ALT (**G**) and plasma total XOR, XO, and XDH activity during the periods of feeding with CDAHFD. *n* = 3 for each group. NAFLD/NASH, nonalcoholic fatty liver disease/steatohepatitis; CDAHFD, choline-deficient, L-amino acid–defined, high-fat diet; NC, normal chow; XDH, xanthine dehydrogenase; XO, xanthine oxidase.

We previously reported that obese conditions upregulate XOR expression in murine adipose tissue (25). Thus, to eliminate the influence of adipose XOR, we examined the causality of the relationship between liver diseases and increased plasma XOR using a mouse model of NAFLD/NASH without obesity. Male mice were fed a choline-deficient, L-amino acid–defined, high-fat diet (CDAHFD) consisting of 0.1% methionine, no choline, and 60 kcal% fat, by which impaired hepatic VLDL-triglyceride secretion rapidly induces hepatic steatosis and fibrosis (26). As shown in Figure 2A, feeding with CDAHFD for up to 6 weeks exhibited time-dependent liver weight gain, despite relative decreases in epididymal white adipose tissue weight (normal chow, NC: 0.45 ± 0.06 g vs. 6 weeks of CDAHFD: 0.39 ± 0.07 g). H&E (Figure 2B, upper panels) and Sirius red staining (Figure 2B, lower panels) of liver tissues supported the development of hepatocellular steatosis during week 1 and NASH-like features of severe hepatic fibrosis during week 6 of CDAHFD. Both hepatic XOR mRNA (Supplemental Figure 1A) and protein (Supplemental Figure 1, B and C) expression levels were significantly upregulated around 2-fold on week 6 of CDAHFD, along with the progression of fibrosis and inflammation in liver (Supplemental Figure 1, A–C). We also observed a steady increase in XO and total XOR activity, but not XDH activity, in liver after feeding with CDAHFD (Supplemental Figure 1D).

Reflecting liver injury, serum AST and ALT markedly increased over time with CDAHFD (Figure 2C), accompanied by increased circulating XOR protein levels detected by Western blotting (Figure 2D). Moreover, plasma XO activity, measured by the HPLC fluorescence detection (HPLC-FLD) method, significantly increased on week 1 of CDAHFD (before the establishment of hepatic fibrosis), reaching more than 10-fold on week 6 (Figure 2E). On the other hand, plasma XDH activity was not affected by the development of NAFLD (Figure 2E). During the periods of CDAHFD feeding, we found significant positive correlations between liver transaminases (serum AST and ALT) and both plasma XO and total XOR activity, but not with plasma XDH activity (Figure 2, F and G). Based on these results, in addition to increased XOR expression in liver, excess leakage of hepatic XOR from the damaged liver was considered to cause markedly high plasma XOR (mainly XO) activity in NAFLD/NASH mice.

### Enhanced purine catabolism in the plasma of NASH mice and in human plasma with highly increased XOR activity.

**Figure 3:**
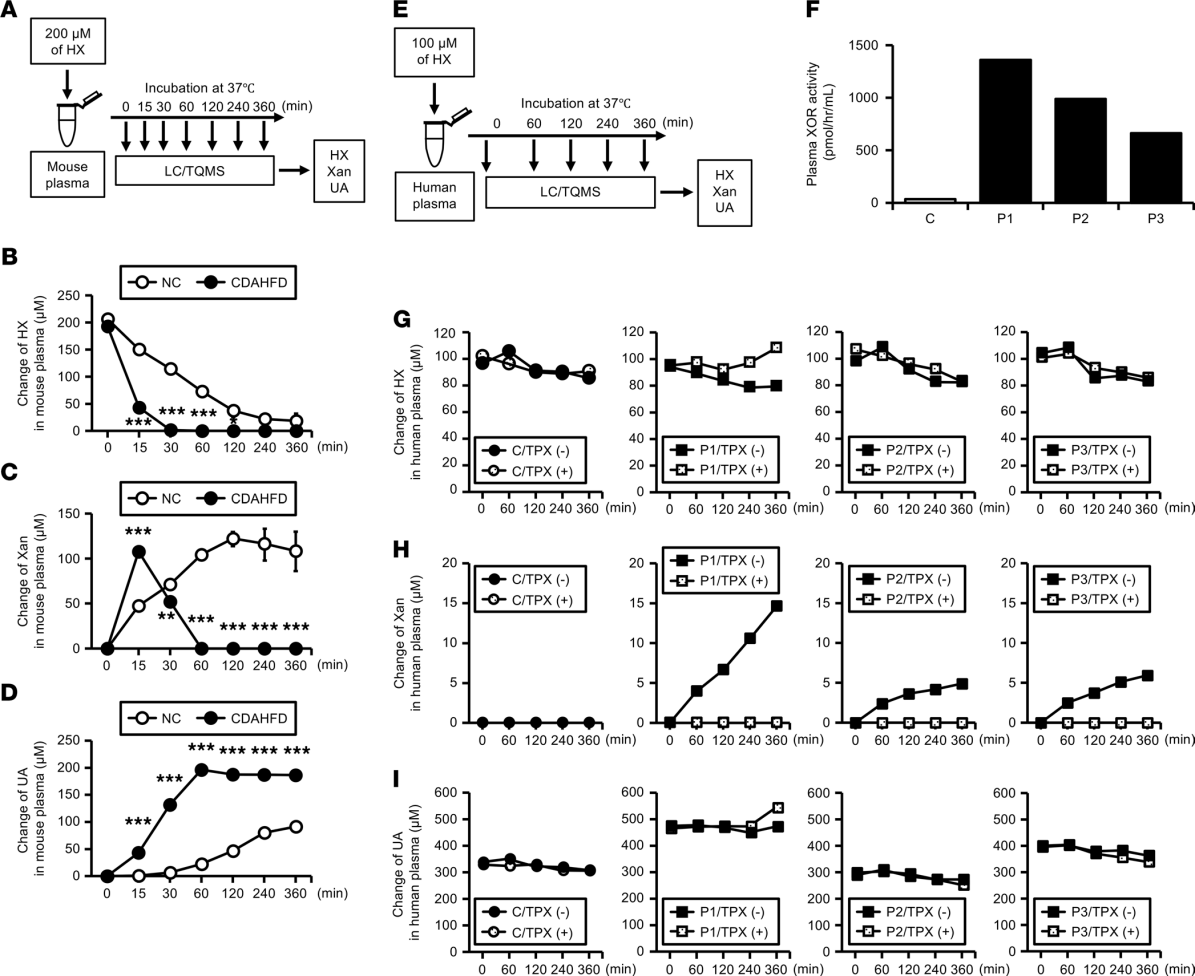
Enhanced purine catabolism in the plasma of NASH mice and of human subjects with highly increased XOR activity. (**A**) Assay for purine metabolic reactions in mouse plasma. HX, Xan, and UA concentrations were measured at each time point, after the addition of 200 μM of HX into plasma samples obtained from male C57BL/6J mice fed NC or a CDAHFD for 6 weeks. (**B**) Changes in HX concentrations. (**C**) Changes in Xan concentrations. Shown are relative changes from 0 minutes. (**D**) Changes in UA concentrations. Shown are relative changes from 0 minutes. White circles = mice fed NC; black circles = mice fed CDAHFD. *n* = 3 for each group. Data are shown as mean ± SEM. *P < 0.05, **P < 0.01, and ***P < 0.001 vs. NC at each time point (2-tailed unpaired *t* test). (**E**) Assay for purine metabolic reactions in human plasma. HX, Xan, and UA concentrations were measured at each time point, after the addition of 100 μM of HX into human plasma samples obtained from a healthy control (abbreviated as C) and patients before bariatric surgery with high XOR activity (P1–P3). These plasma samples were pretreated with or without 10 μM TPX before the assay. (**F**) Plasma XOR activity in each plasma sample used in this assay. XOR activity was measured using the LC/TQMS method. (**G**–**I**) Changes in HX (**G**), Xan (**H**), and UA (**I**) concentrations. Black circles = plasma from a healthy control without TPX; open circles = plasma from a healthy control pretreated with TPX; black squares = plasma from patients without TPX; open squares = plasma from patients pretreated with TPX. HX, hypoxanthine; Xan, xanthine; UA, uric acid; TPX, topiroxostat.

Next, we asked whether increased XOR activity in NASH mice alters purine catabolism in the plasma. As described in Methods, the conversions of hypoxanthine to xanthine and uric acid were assessed after the addition of hypoxanthine (200 μM), as a substrate for XOR, into plasma obtained from mice fed NC or CDAHFD for 6 weeks (Figure 3A). Changes in hypoxanthine, xanthine, and uric acid concentrations are provided in Figure 3, B–D, respectively. In plasma of CDAHFD-fed mice (indicated by black circles), hypoxanthine rapidly decreased to undetectable levels at 30 minutes (Figure 3B), and then xanthine peaked at 15 minutes and subsequently diminished at 60 minutes (Figure 3C). Lastly, hypoxanthine added to the plasma was fully converted to uric acid within 60 minutes, reaching a plateau of 200 μM (Figure 3D). Contrary to this, in plasma from mice fed NC diet (indicated by white circles), hypoxanthine and xanthine gradually decreased and increased, respectively, over 360 minutes, and uric acid production remained below 100 μM even at 360 minutes (Figure 3, B–D). These results suggest that high plasma XOR activity in NASH mice accelerated the purine catabolic rate in the circulation.

We also performed a similar assay using human plasma obtained from a healthy control and patients before bariatric surgery who had high plasma XOR activity (C and P1–P3, respectively; Figure 3E). XOR activity levels measured by the LC/TQMS method in each plasma sample used in this experiment are shown in Figure 3F. In the plasma of patients with high XOR (indicated by black squares), when 100 μM of hypoxanthine was added into the plasma, the hypoxanthine concentration gradually decreased over 360 minutes (Figure 3G), which resulted in an increased concentration of xanthine (Figure 3H). Such xanthine production was blocked by pretreatment with 10 μM of topiroxostat, a nonpurine selective inhibitor of XOR (indicated by open squares; Figure 3H). There was no increase of xanthine in the plasma from a control subject, regardless of the presence of topiroxostat (indicated by dot circles and black circles; Figure 3H). Similar results of no increase in the xanthine concentration were obtained for 2 other healthy controls, whose plasma XOR activity was 28.7 pmol/h/mL and 33.7 pmol/h/mL, respectively (data not shown). Uric acid levels were not changed in all plasma samples of patients and controls, possibly due to the high baseline concentrations (Figure 3I). These results suggest that XOR-dependent purine catabolism, at least hypoxanthine to xanthine, also occurred in the human blood when plasma XOR activity increased to a certain extent.

### Amelioration of vascular neointima formation in NASH mice by XOR inhibition.

**Figure 4:**
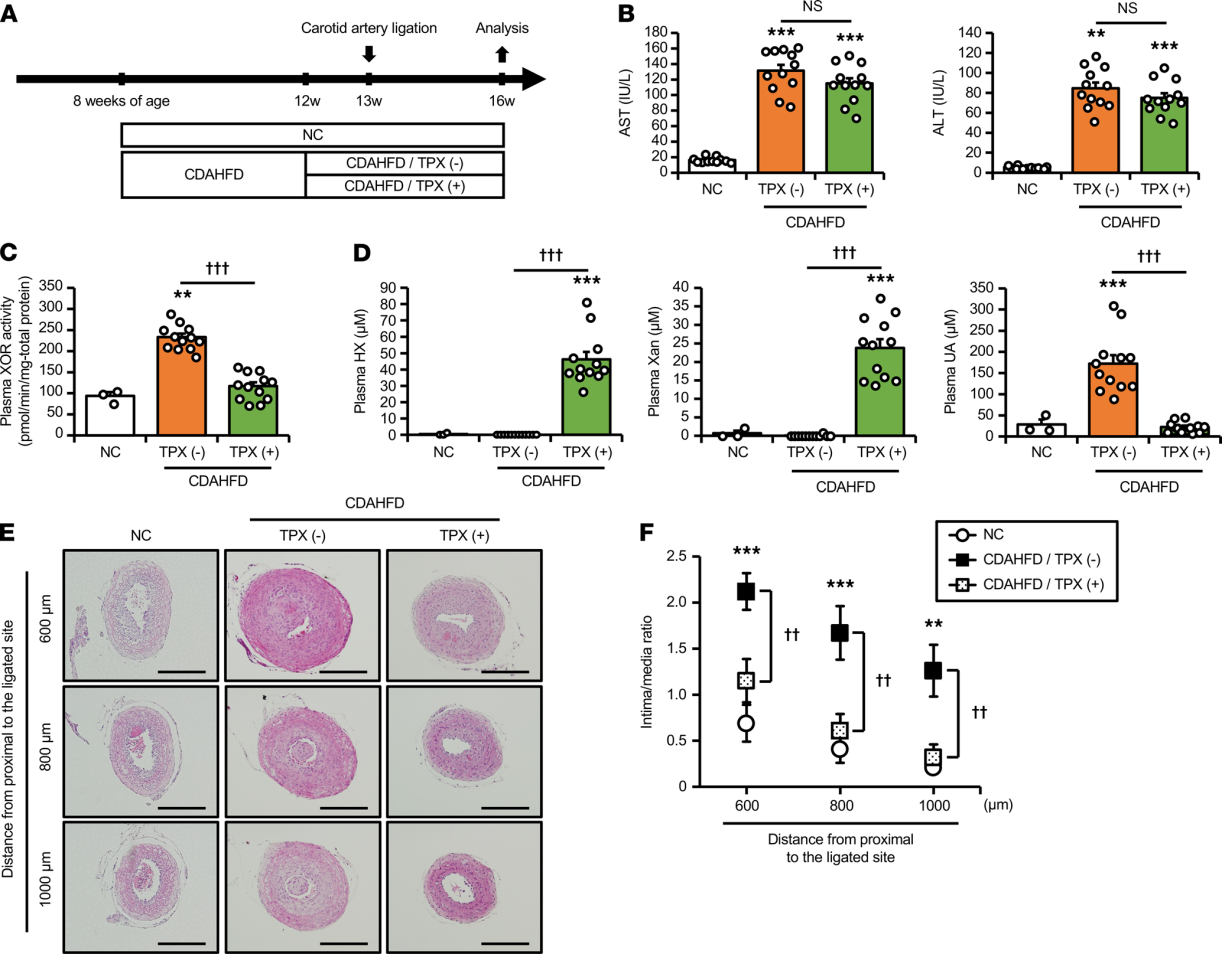
Suppressive effects of topiroxostat, a selective XOR inhibitor, on neointima formation induced by carotid artery ligation in NASH mice. (**A**) Male C57BL/6J mice were divided into 3 groups: fed NC or fed a CDAHFD treated with or without TPX (1 mg/kg/d). Unilateral carotid artery ligation was performed to induce neointima formation 5 weeks after feeding with each diet. TPX was administered from 1 week prior to carotid artery ligation, and all mice were examined 3 weeks after surgery. (**B**) Serum AST and ALT in each group. *n* = 11 for NC and *n* = 12 for CDAHFD/TPX (-) and CDAHFD/TPX (+). (**C**) Plasma XOR activity measured by the LC/TQMS method in each group. *n* = 3 for NC and *n* = 12 for CDAHFD/TPX (-) and CDAHFD/TPX (+). (**D**) Plasma concentrations of HX, Xan, and UA in each group. *n* = 3 for NC and *n* = 12 for CDAHFD/TPX (-) and CDAHFD/TPX (+). (**E**) Representative H&E-stained sections of ligated carotid arteries obtained from each group. Scale bars: 200 μm. (**F**) Quantification of the intima/media ratio at 600 μm, 800 μm, and 1000 μm from proximal to the ligated site. *n* = 11 for NC and *n* = 12 for CDAHFD/TPX (-) and CDAHFD/TPX (+). Data are shown as mean ± SEM; **P < 0.01 and ***P < 0.001 vs. NC and ^††^*P* < 0.01, and ^†††^*P* < 0.001. NS (1-way ANOVA with Tukey’s post hoc test). I/M, intima/media.

Based on both human and mouse studies, high plasma XOR activity is directly associated with liver disease conditions such as NAFLD/NASH. Accumulating clinical data suggest that NAFLD/NASH increases the risk of CVDs independent of established cardiovascular risk factors (27–29). This background prompted us to conduct further experiments to elucidate whether excess plasma XOR activity participates in such crosstalk between NAFLD/NASH and CVD progression. As shown in Figure 4A, mice were divided into 3 groups: mice that were fed NC (control) or CDAHFD treated with, CDAHFD/TPX (+), or without, CDAHFD/TPX (-), topiroxostat and then subjected to unilateral carotid artery ligation to induce neointima formation. In the CDAHFD/TPX (+) group, topiroxostat was administered 1 week prior to carotid artery ligation, and all mice were analyzed 3 weeks after surgery (8 weeks after feeding with NC or CDAHFD; Figure 4A).

The administration of topiroxostat did not affect food intake (Supplemental Figure 2A), body weight (Supplemental Figure 2B), and liver weight gain (Supplemental Figure 2C) under CDAHFD feeding. In liver, increased XOR activity observed in NASH mice fed CDAHFD was significantly suppressed by topiroxostat (Supplemental Figure 3A). The amount of hypoxanthine in liver was higher in the CDAHFD/TPX (-) group than in the control group, and it was further increased in the CDAHFD/TPX (+) group (Supplemental Figure 3B). Hepatic xanthine and uric acid levels were also significantly increased in the CDAHFD/TPX (-) group, and they were markedly reduced by topiroxostat (Supplemental Figure 3B). Although topiroxostat inhibited both XOR activity and XOR-dependent purine catabolism in liver, the extent of hepatic steatosis and fibrosis was not different between the CDAHFD/TPX (-) and CDAHFD/TPX (+) groups (Supplemental Figure 3, C and D). Topiroxostat did not affect CDAHFD-induced increases in liver α–smooth muscle actin, collagen type I, and TNF-α mRNAs (Supplemental Figure 3E) and αSMA protein (Supplemental Figure 3, F and G) expression, as well.

Consistent with the minimal effect of topiroxostat on hepatic damage, serum AST and ALT were not different between the CDAHFD/TPX (-) and CDAHFD/TPX (+) groups (Figure 4B). Topiroxostat administration diminished the high plasma XOR activity induced by NASH (Figure 4C). Plasma concentrations of purine metabolites are shown in Figure 4D. Although plasma hypoxanthine and xanthine were barely detectable in the control and the CDAHFD/TPX (-) groups, they were increased to around 50 μM and 25 μM, respectively, in the CDAHFD/TPX (+) group. Plasma uric acid concentrations were markedly increased in the CDAHFD/TPX (-) group, but those in the CDAHFD/TPX (+) group were decreased to similar levels of the control group. This suggested that topiroxostat almost completely inhibited XOR-mediated purine catabolism in the circulation. Besides, these results disclosed another point of interest in that hypoxanthine and xanthine, substrates for XOR (XO), were potentially abundant in the bloodstream of NASH mice, which is generally masked by their high plasma XOR activity.

Next, neointima formation induced by carotid artery ligation was evaluated to investigate the significance of high plasma XOR associated with NAFLD/NASH on vascular injury. Regarding serum lipid profiles, triglyceride (TG; Supplemental Figure 2D) and nonesterified fatty acids (NEFAs; Supplemental Figure 2E) levels were not different between the CDAHFD/TPX (-) and CDAHFD/TPX (+) groups. Serum total cholesterol levels were also unchanged between the 2 groups (Supplemental Figure 2F), which were significantly lower than the control group. Of note, NASH mice fed CDAHFD exhibited marked progression of neointima formation (Figure 4E), demonstrated by significant increases in the intima-to-media ratio compared with the control group at all distances (600 μm, 800 μm, and 1000 μm) from proximal to the ligated site (Figure 4F). In contrast, topiroxostat significantly attenuated neointima formation in NASH mice to similar levels observed in the control group (Figure 4, E and F). These results suggest that XOR inhibition in NASH mice had a significant effect on preventing neointimal proliferation induced by carotid artery ligation.

### Effects of liver-derived XOR on vascular smooth muscle cell proliferation and dedifferentiation.

**Figure 5:**
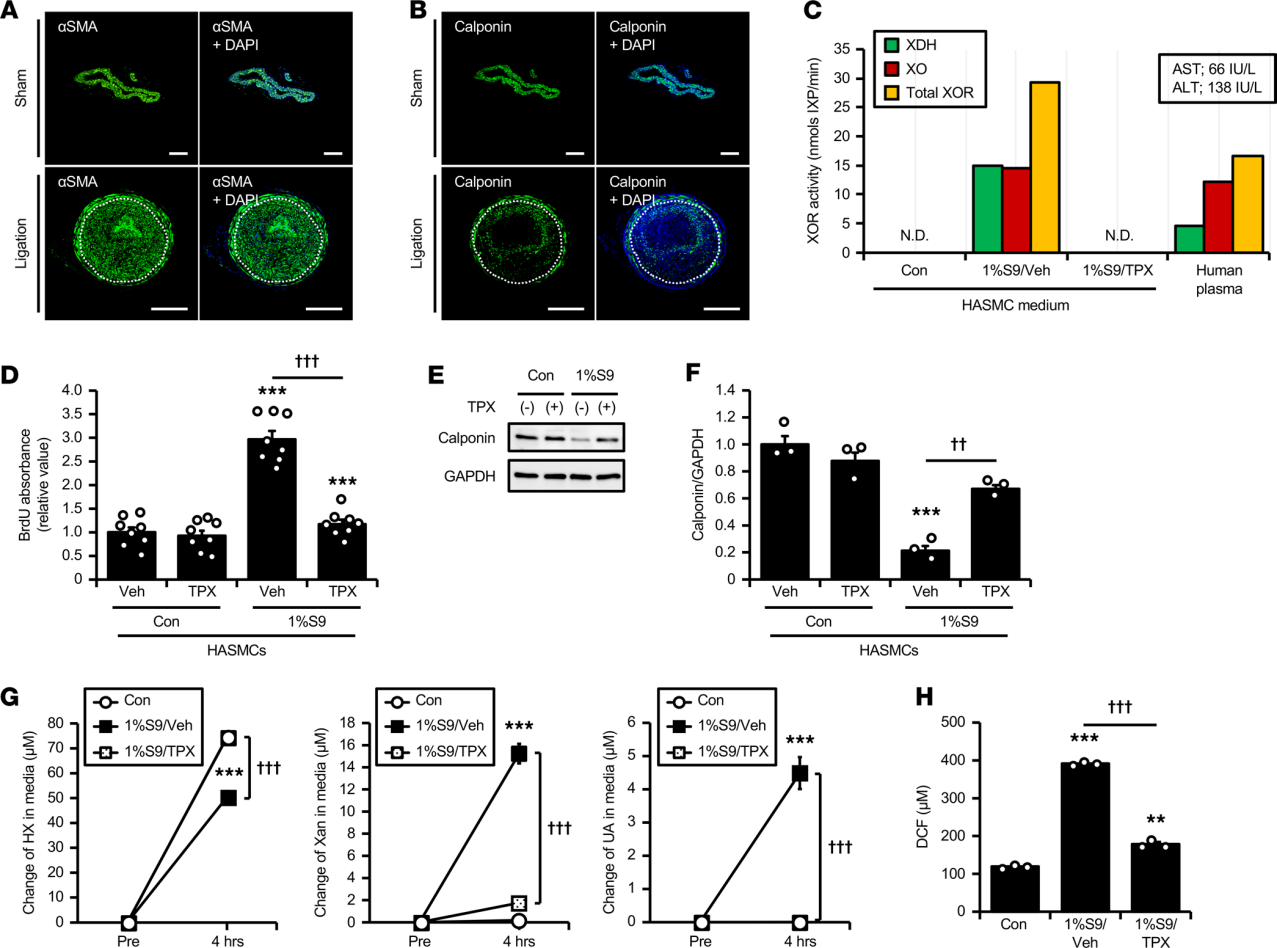
Effects of liver S9–derived XOR on proliferation and dedifferentiation in HASMCs. (**A** and **B**) Unilateral carotid artery ligation was performed on male C57BL/6J mice 5 weeks after a CDAHFD feeding. Then vessels were collected 3 weeks after surgery. Representative immunofluorescence images for αSMA (green; **A**) or calponin (green; **B**) with DAPI-stained nuclei (blue) of sham-operated (upper panels) and ligated arteries (lower panels). The border between media and neointima was indicated by the dotted line. Scale bar: 150 μm. (**C–F**) HASMCs were incubated with or without 1% human liver S9 compartment (S9) in the presence or absence of 10 μM TPX. (**C**) XDH, XO, and total XOR activity in cell culture media and human plasma of an obese patient with liver dysfunction. The HPLC-FLD method was used to distinguish between XO activity and total XOR (XO plus XDH) activity. (**D**) The relative incorporation of BrdU. *n* = 8 for each group. (**E**) Immunoblots for calponin and GAPDH. (**F**) Relative protein levels of calponin normalized to GAPDH. *n* = 3 for each group. (**G** and **H**) HUVECs were incubated with or without 1% S9 in the presence or absence of 10 μM TPX for 4 hours. (**G**) Changes in HX, Xan, and UA concentrations in the culture media. White circles = control; black squares = 1% S9 with vehicle (Veh); open squares = 1% S9 with TPX. *n* = 3 for each group. (**H**) ROS production in the culture media measured by a fluorogenic probe, DCFH-DiOxyQ, using DCF standard. *n* = 3 for each group. Data are shown as mean ± SEM; **P < 0.01 and ***P < 0.001 vs. control (Con, without 1% S9), and ^††^*P* < 0.01 and ^†††^*P* < 0.001 (1-way ANOVA with Tukey’s post hoc test). ND, not detected; HASMCs, human arterial smooth muscle cells; αSMA, α–smooth muscle actin; DCFH-DiOxyQ, dichlorodihydrofluorescin DiOxyQ.

Vascular smooth muscle cells (VSMCs) dedifferentiate from the contractile to the synthetic phenotype under pathological conditions such as atherosclerosis (30, 31). As shown in Figure 5A, immunostaining demonstrated that progressively developed neointima of NASH mice predominantly consisted of αSMA-positive cells. In contrast, expression of calponin, a marker of contractile SMCs, was markedly reduced in αSMA-positive neointima (Figure 5B). This cellular pattern indicates that dedifferentiated SMCs played a central role in the process of neointima formation. Further in vitro experiments were performed to examine the effects of liver-derived XOR on SMC proliferation and dedifferentiation, using the human liver S9 compartment as a source of hepatic XOR (32). We confirmed that S9 diluted to 1% had equivalent XO and total XOR activity to those in plasma of the patient before bariatric surgery with liver dysfunction (the same sample as P1 in Figure 3), whose serum AST and ALT levels were 66 IU/L and 138 IU/L, respectively (Figure 5C). Such relatively high XOR activity of 1% S9 decreased to an undetectable level by topiroxostat (Figure 5C). Under serum-free conditions, incorporation of BrdU was significantly increased by incubating human arterial smooth muscle cells (HASMCs) with 1% S9, which was suppressed by treatment with topiroxostat (Figure 5D). Exposure of xanthine or uric acid, byproducts of XOR, itself did not alter BrdU incorporation (Supplemental Figure 4, A and B). In addition, 72 hours culture with 1% S9 significantly decreased calponin protein, whereas such effects of S9 were also attenuated by topiroxostat (Figure 5, E and F). From these data, it was supposed that liver-derived XOR promoted SMC proliferation and dedifferentiation in its enzymatic activity-dependent manner.

Finally, given the current concept that XOR is involved in vascular endothelial injury (19), we examined the effects of liver-derived XOR on human ECs. Reflecting low expression level of the *XDH* gene and XOR activity in human umbilical vein endothelial cells (HUVECs; data not shown), only hypoxanthine was measurable in cell lysates, and both xanthine and uric acid were below the detectable range (Supplemental Figure 4C). Regardless of the presence of topiroxostat, incubation with 1% S9 for 4 hours did not alter the intracellular amount of these 3 purine metabolites, suggesting that XOR derived from S9 had no intracellular effect (Supplemental Figure 4C). As shown in Figure 5G, we detected high levels of hypoxanthine secreted from HUVECs in the control culture media without S9 (indicated by white circles), reaching over 70 μM after 4 hours of incubation. On the other hand, there was no increase in xanthine or uric acid levels (Figure 5G). When HUVECs were incubated with 1% S9 (indicated by black squares), activation of purine catabolism was observed in the culture medium, represented by the reduction of hypoxanthine and the production of xanthine and uric acid (Figure 5G), which was accompanied by ROS production in the medium (Figure 5H). These catabolic reactions and ROS generation induced by 1% S9 were completely abolished by topiroxostat (indicated by open squares; Figure 5, G and H). Under these conditions, mRNA levels of VCAM-1 and E selectin were significantly increased by the incubation with 1% S9 for 4 hours, whereas topiroxostat suppressed these effects of S9 (Supplemental Figure 4D). Xanthine or uric acid did not alter these adhesion molecules’ expression levels (Supplemental Figure 4, E and F).

## Discussion

The major findings of the present study are as follows: (a) Changes in plasma XOR activity between before and after bariatric surgery were closely associated with those in serum AST and ALT but not in BMI. (b) Nonobese NASH mice fed CDAHFD exhibited markedly increased plasma XOR activity, accompanied by enhanced purine catabolism in plasma. (c) The in vivo administration of the XOR inhibitor to NASH mice markedly attenuated the development the development of neointimal proliferation induced by carotid artery ligation. (d) In the in vitro experiments, human liver S9–derived XOR accelerated proliferation of SMCs with reduction of calponin expression and induced ROS production by catabolizing hypoxanthine released form ECs in its enzymatic activity–dependent manner.

An initial objective of this project was to determine distinct pathological features that are clinically associated with increased plasma XOR activity. In our follow-up study on obese patients who underwent bariatric surgery, significant reductions in plasma XOR activity were observed 1 year after the surgery (*P* < 0.001), together with the marked weight loss (the mean value of –22.1 kg). It is somewhat surprising that changes in plasma XOR activity during this period were not correlated with those in body weight or BMI because previous cross-sectional (22, 23) and longitudinal (33) studies demonstrated the relationships between plasma XOR and BMI. Instead, only changes in serum liver transaminases (AST and ALT) had significant positive correlations with those in plasma XOR (Figure 1 and Supplemental Table 4). These differing results may be explained by differences in baseline characteristics. Compared with those described in the general population (22, 23, 33), baseline levels of plasma XOR activity in our patients were distributed in a wide range of 16.2–1360 pmol/h/mL, presumably reflecting a higher prevalence of liver dysfunction with severe obesity. Taken together with our previous findings that the expression levels of human XOR were far lower in adipose tissue than in liver and small intestine (11), we conclude that plasma XOR activity increased mainly according to the degree of liver dysfunction.

Importantly, we confirmed high plasma XOR activity also in nonobese NAFLD/NASH mice fed CDAHFD. Regarding XOR in liver, we and others demonstrated increased hepatic XOR activity in high-fat diet–induced obesity and genetically obese mice (25, 34, 35). Consistent with these previous observations, mice fed CDAHFD exhibited time-dependent upregulation of hepatic XOR at the translational level, along with the development of fibrosis and inflammation in liver. Furthermore, in addition to changes in liver, a marked increase in plasma XOR activity was observed from as early as 1 week after CDAHFD feeding before the establishment of NASH-like features of hepatic fibrosis. A strong positive correlation was also found between liver transaminases (both serum AST and ALT) and plasma XOR activity during the progression of NAFLD/NASH in this mouse model. These results strongly suggest that plasma XOR activity begins to increase at the early stage of NAFLD, probably followed by increased leakage of hepatic XOR from the damaged liver. This causal relationship between NAFLD/NASH conditions and high plasma XOR activity should also apply to humans, in whom XOR is more exclusively produced in liver (11).

One of the most important issues emerging from these findings is whether such an increase in plasma XOR has pathological significance beyond being a biomarker of hepatic damage. In recent years, several clinical investigations revealed that NAFLD/NASH increases the risk of atherosclerosis and coronary artery disease independent of traditional cardiovascular risk factors (27–29). Although the underlying mechanism of this association has not been fully elucidated, several factors related to NAFLD/NASH, including insulin resistance, dietary intake, altered lipid metabolism, the gut microbiome, and the proinflammatory state, are considered to play roles in the pathogenesis of atherosclerosis (36–38). Supporting the clinical evidence mentioned above, NASH mice fed CDAHFD exhibited significantly augmented neointimal proliferation induced by carotid artery ligation (*P* < 0.001).Topiroxostat is reported to inhibit plasma XOR activity more effectively than other XOR inhibitors, allopurinol and febuxostat, in both mice (39) and humans (40). Furthermore, XOR inhibition by topiroxostat reversed both increased plasma XOR activity and the progression of neointima formation in NASH model mice of the present study to similar levels observed in control mice independent of liver dysfunction (Figure 4, E and F). These results suggested an impact of excess XOR release from the damaged liver as a key mediator of NAFLD/NASH-related atherosclerosis.

In the development of atherosclerosis, local environmental cues such as inflammatory stimuli induce the phenotypic dedifferentiation of VSMCs from the quiescent contractile type to the synthetic type, which results in proliferation and migration of SMCs from the media into the intima (30, 31, 41). In this regard, one of the major findings of this study is the effect of liver-derived XOR to induce proliferative phenotypic switch of VSMCs. As expected, immunostaining revealed that αSMA-positive/calponin-negative dedifferentiated SMCs were primarily involved in neointima formation in NASH mice (Figure 5, A and B). A previous study clearly showed that XO exerted a distinct effect on proliferation of rat VSMCs through O_2_^–^ (42). Similarly, human liver S9 compartment, which was adjusted to have XOR activity equivalent to that observed in human plasma, stimulated proliferation, and dedifferentiation of human VSMCs, and these effects of S9 were highly dependent on the enzymatic activity of XOR but not on xanthine or uric acid, the byproducts of XOR (Figure 5, D–F). Collectively, our present data propose the possible association between pathological SMC phenotypic modulation and excess XOR from damaged liver.

In both mice and humans, circulating XOR that increased under NAFLD/NASH conditions was predominantly in the XO form. Because the involvement of XO in pathological processes is well described by its potential to generate ROS (18–20), it is important to know whether purine catabolism indeed occurs in the circulation. In this context, we demonstrated for the first time to our knowledge that hypoxanthine was converted to xanthine and uric acid in mouse plasma, and these reactions were markedly augmented in NASH mice fed CDAHFD. We also confirmed that XOR activity highly increased in patients before bariatric surgery, whose plasma exhibited the potential to catabolize hypoxanthine to xanthine. Regarding its substrate, we previously reported hypoxanthine secretion from human adipocytes (11). In addition, a recent clinical study on the general population demonstrated that the plasma hypoxanthine concentration is positively associated with BMI, suggesting increased hypoxanthine release from obese adipose tissue (43). Based on this study, human ECs are also considered as a potential source of hypoxanthine. Due to the absence of intracellular XOR, we detected a large amount of hypoxanthine secreted from HUVECs into the culture media (Figure 5G). Together with previous observations that XOR can bind to endothelial cell membranes (16, 17), hypoxanthine supplied from the circulation or released from vascular ECs could have been subsequently catabolized on the cell surface of ECs, especially in individuals with particularly high plasma XOR activity. We also detected significant ROS production induced by XOR-mediated hypoxanthine catabolism in cell culture medium of HUVECs (Figure 5H). This local ROS generation on the vasculature, rather than the XOR byproducts, may lead to endothelial proinflammatory responses, represented by the upregulation of endothelial adhesion molecules and the proliferation and dedifferentiation of SMCs, which synergistically accelerate vascular remodeling.

Several studies have provided evidence that XOR is involved in macrophage activation. XOR inhibition in macrophages was reported to prevent foam cell formation (44), cholesterol crystal–induced ROS generation (45), and the NLRP3 inflammasome (46). At least partly through these suppressive effects on macrophages, XOR inhibitors, febuxostat and allopurinol, might attenuate atherosclerotic plaque formation in apolipoprotein E–KO mice (44, 45). Thus, it is also possible that increased circulating XOR in NAFLD/NASH activates macrophages in the vasculature, leading to the progression of atherosclerosis. However, these previous investigations focused on XOR expressed in macrophages; therefore, it remains to be elucidated whether XOR in the circulation also activates macrophages.

To date, clinical evidence for the effects of XOR inhibition on atherosclerosis is controversial. In 2 randomized trials, allopurinol attenuated the progression of carotid intima-media thickness in patients with type 2 diabetes, asymptomatic hyperuricemia, and recent ischemic stroke (8, 9). On the other hand, a recent randomized controlled trial of Japanese patients with asymptomatic hyperuricemia reported that 24-month treatment with another XOR inhibitor, febuxostat, did not delay carotid atherosclerosis progression (10). According to the present study, patients with NAFLD/NASH, whose plasma XOR is typically high, may be the subjects most likely to benefit from XOR inhibitors. However, phenomena observed using mouse models do not necessarily apply to humans due to marked differences between rodents and humans in the tissue distribution of XOR, plasma XOR activity, and purine metabolism. To clarify whether XOR inhibitors can prevent or delay cardiovascular complications associated with increased plasma XOR activity, as in mice, large-scale studies enrolling patients with NAFLD/NASH will be required in the future.

The working hypothesis from the present study could be summarized in Supplemental Figure 5. We propose that high plasma XOR activity was directly induced by liver disease conditions, such as NAFLD/NASH, and accelerated purine catabolism in the circulation, which may be related to the development of vascular neointimal proliferation. This study adds insight into the understanding of increased plasma XOR activity and its causal roles in the pathogenesis of vascular injury. XOR inhibitors may have the potential as a therapeutic option for preventing atherosclerotic diseases in patients with NAFLD/NASH.

## Methods

### Animal study.

Male C57BL/6J mice were purchased from CLEA Japan Inc. For the nonobese NAFLD/NASH model (26), mice were fed CDAHFD (Research Diets) from 8 weeks of age. Mice were maintained at 22°C under a 12-hour light/12-hour dark cycle (lights on from 8 am to 8 pm).

For carotid artery ligation, mice were divided into 3 groups: the first group was fed NC, and the other 2 groups were fed CDAHFD supplemented with or without topiroxostat (Sanwa Kagaku), a nonpurine selective inhibitor of XOR. At 4 weeks after CDAHFD feeding, topiroxostat (1 mg/kg/d) was administered to mice by the drug-admixed food method. At 5 weeks after feeding with NC or CDAHFD (1 week after the administration of topiroxostat), mice were exposed through a small midline incision in the neck. The left common carotid artery was then ligated entirely with a 6-0 silk thread just proximal to the carotid bifurcation to disrupt blood flow (47, 48). Carotid artery occlusion typically results in inflammatory vessel changes, shrinkage, neointima formation, and narrowing of the vascular lumen. Three weeks after ligation, the left (ligated side) common carotid arteries were excised and subjected to analyses. In all experiments, mice were anesthetized by i.p. injection of a mixture of medetomidine (0.3 mg/kg body weight), midazolam (4 mg/kg body weight), and butorphanol tartrate (5 mg/kg body weight) and analyzed.

### Histological analysis.

Livers and the left (ligated side) common carotid arteries were excised from mice after transcardiac perfusion with ice-cold saline. Isolated tissues were fixed with 4% paraformaldehyde and embedded in paraffin. Subsequently, samples were cut into 2 μm thick sections and mounted on glass slides. Sections were stained with H&E. Quantification of vascular remodeling at 600, 800, and 1000 μm proximal to the ligated site was performed by measuring the area of neointima and media using NIH ImageJ software.

Liver fibrosis was evaluated by Sirius red staining. Paraffin-embedded liver sections were incubated for 90 minutes at room temperature with saturated picric acid containing 0.1% Fast Green FCF and 0.1% Direct Red (ScyTek Laboratories). To calculate Sirius red–positive areas, 3 randomly obtained sections (×100) using a BZ-X700 microscope (Keyence) were analyzed for each mouse. The same color tone in all the images was converted to gray scale and measured at the same threshold using ImageJ software.

### Immunofluorescence staining.

Paraffin-embedded sections of carotid arteries were blocked with 3% FBS at room temperature and then incubated with the following primary antibodies overnight under 4°C: rabbit anti-αSMA (1:100; ab5694, Abcam) and rabbit anti-calponin (1:100; ab46794, Abcam) in PBS containing 1% FBS. Biotinylated IgG (1:200; BA-1000, Vector Laboratories) was used as secondary antibody, followed by detection with FITC-conjugated streptavidin. Cell nuclei were counterstained with DAPI (Invitrogen). Microscopy analysis was performed using an Olympus FV3000 confocal laser scanning microscope system (Olympus).

### Cell culture.

HASMCs (Lonza) were maintained in SmGM2 medium (Lonza) with 5% FBS, and cells with 4–6 passages were used for experiments. We used pooled postmitochondrial supernatant fractions, a mixture of microsomes and cytosol, from homogenized human livers (S9 compartment, Sekisui Medical) as a source of hepatic XOR (32). After purification by the Sephadex G25 column (PD MiniTrap G25, GE Healthcare), S9 was diluted to 1% in the cell culture media. After 24 hours of serum starvation, HASMCs were incubated with these agents under serum-free conditions for 72 hours by exchanging the medium every 24 hours. Proliferation of HASMCs was quantified by the BrdU assay (MilliporeSigma), according to the manufacturer’s protocol. Briefly, cells were incubated with 1% S9 in the presence or absence of 10 μM of topiroxostat for 24 hours and then labeled with BrdU for additional 18 hours at 37°C. The absorbance of BrdU-labeled cells was measured by Microplate Reader SH-9000 (Corona Electric). HUVECs (Kurabo) from 5 to 7 passages were maintained in EBM-2 medium (Kurabo) with 2% FBS, 100 units/mL of penicillin, and 100 μg/mL of streptomycin at 37°C in a humidified atmosphere of 5% CO_2_. HUVECs were grown to subconfluence in collagen-coated, 12-well plates and incubated with 1% S9 in the presence or absence of 10 μM of topiroxostat for 4 hours. ROS production in the culture media was measured using the OxiSelect In Vitro ROS/RNA Assay Kit (Cell BioLabs) according to the manufacturer’s protocol. This assay allows the measurement of total ROS/reactive nitrogen species by dichlorodihydrofluorescin DiOxyQ. Samples were quantified fluorometrically against the dichlorodihydrofluorescin standard.

### Measurements of XOR activity.

XOR activity was quantified using LC/TQMS by detecting the production of [^13^C_2_, ^15^N_2_]-uric acid from [^13^C_2_, ^15^N_2_]-xanthine as described previously (21). Briefly, liver tissues were homogenized in PBS (pH 7.4) containing protease inhibitor cocktail (Roche) and centrifuged at 20,000*g* at 4°C for 10 minutes. These homogenates or plasma samples were added to Tris buffer (pH 8.5) mixture containing [^13^C_2_, ^15^N_2_]-xanthine, NAD^+^, and oxonate (uricase inhibitor), followed by incubation at 37°C for 30 minutes. Then methanol containing [^13^C_2_, ^15^N_2_]-uric acid (the internal standard) was added into the mixture and centrifuged at 3000*g* at 4°C for 15 minutes. Subsequently, the supernatant was dried by a centrifugal evaporator. The residues were reconstituted with 150 μL of distilled water, filtered through an ultrafiltration membrane, and measured using LC/TQMS, which comprised a Nexera LC system (Shimadzu) and a QTRAP 4500 Mass Spectrometry System (SCIEX) equipped with an ESI interface. Calibration standard samples of [^13^C_2_, ^15^N_2_]-uric acid were also measured, and production levels were calculated from the calibration curve.

### Measurements of XOR activity discriminating between XO and XDH.

XO and total XOR (XO plus XDH) activity were respectively measured using the HPLC-FLD method (25), detecting the conversion of pterin to isoxanthopterin. Tissues were homogenized in RIPA buffer containing protease inhibitor (complete mini, Roche Diagnostics) and centrifuged at 20,000*g* for 15 minutes at 4°C. To measure XO activity, protein lysates, plasma or cell culture media were incubated at 37°C for 180 minutes with 50 μM of pterin, followed by treatment with 2% perchloric acid. The chromatographic system was a NANOSPACE SI-2 (Shiseido) using a Capcell Pak C18 MGIII column (Shiseido). Separation was performed at 40°C using 20 mM potassium phosphate buffer (pH 2.4), and fluorescence intensity was measured at excitation/emission = 345 nm/410 nm every 5 minutes. To measure total XOR (XO plus XDH) activity, samples were incubated with 25 μM of pterin and 200 μM of methylene blue under the same conditions. The reaction rate was determined from the slope of the intensity versus time plots. The known amount of isoxanthopterin was used for quantification. Values are expressed as nanomoles of isoxanthopterin/minute.

### Measurements of purine metabolites.

Liver tissues and cells were homogenized in PBS containing protease inhibitor cocktail and centrifuged at 20,000*g* for 20 minutes at 4°C. These homogenates, plasma, and cell culture media were added to methanol containing [^13^C_3_, ^15^N_2_]-hypoxanthine, [^13^C_2_, ^15^N_2_]-xanthine, and [^13^C_2_, ^15^N_2_]-uric acid as the internal standards, and then centrifuged at 3000*g* at 4°C for 15 minutes. The supernatant (40 μL) was diluted with distilled water (160 μL), and concentrations of hypoxanthine, xanthine, and uric acid were measured using LC/TQMS (Nexera, Shimadzu/QTRAP 4500, SCIEX).

### Assay for purine catabolic reactions in mouse and human plasma.

To assess purine catabolic reactions in mouse and human plasma, hypoxanthine conversions to xanthine and uric acid were evaluated as follows. The reaction was started by adding hypoxanthine into each plasma sample (200 μM for mice and 100 μM for human in the presence or absence of 10 μM of topiroxostat) and then these mixtures were immediately incubated at 37°C. At each time point, 10 μL of plasma was added to 300 μL of methanol containing [^13^C_3_, ^15^N_2_]-hypoxanthine, [^13^C_2_, ^15^N_2_]-xanthine, and [^13^C_2_, ^15^N_2_]-uric acid as the internal standards and centrifuged at 15,000*g* at 4°C for 10 minutes. The supernatant (200 μL) was subjected to analysis for the measurement of hypoxanthine, xanthine, and uric acid levels by the same method described above.

### Measurement of blood parameters in mice.

Serum AST and ALT were measured by the Transaminase Cii Test (Wako Pure Chemical). Serum total cholesterol, TG, and NEFA were measured by the Cholesterol E Test (Wako Pure Chemical), TG E Test (Wako Pure Chemical), and NEFA C Test (Wako Pure Chemical), respectively.

### Immunoblotting.

Frozen liver tissues and cells were lysed in RIPA buffer (50 mM Tris-HCl at pH 7.4, 150 mM NaCl, 1% Triton X-100, 0.5% sodium deoxycholate, 0.1% SDS, 1 mM EDTA, and 10 mM NaF) containing protease inhibitor. Protein lysates, obtained after centrifugation at 15,000*g* at 4°C for 30 minutes, were boiled with sample buffer (2% SDS, 50 mM Tris-HCl, 10% glycerol, and 6.6% 2-mercaptoethanol) at 98°C for 5 minutes. The same amount of protein samples and diluted serum (precisely equivalent to 0.05 μL of the serum) was subjected to a 4%–20% gradient SDS-PAGE (Bio-Rad). The following primary antibodies were used: mouse anti-XOR (1:1000; sc-398548, Santa Cruz Biotechnology), rabbit anti-αSMA (1:1000; ab5694, Abcam), rabbit anti-calponin (1:1000; ab46794, Abcam), rabbit anti–α-Tubulin (1:1000; 11H10, Cell Signaling Technology), and rabbit anti-GAPDH (1:2000; 14C10, Cell Signaling Technology). Chemiluminescence signals were visualized by ChemiDoc Touch and quantitated using Image Lab software (Bio-Rad).

### Quantitative RT-PCR.

Isolation of total RNA and synthesis of cDNA was performed as described previously (49). Real-time quantitative RT-PCR was performed with the ViiA 7 Real-Time PCR System (Life Technologies) using Power SYBR Green PCR Master Mix (Life Technologies). Primers used in this study were as follows: mouse XOR, forward 5′-ATGACGAGGACAACGGTAGAT-3′ and reverse 5′-TCATACTTGGAGATCATCACGGT-3; mouse αSMA, forward 5′-TTTCATTGGGATGGAGTCAG-3′ and reverse 5′-TCCTTCCTGATGTCAATATCAC-3; mouse collagen type I, forward 5′-GTCCCAACCCCCAAAGAC-3′ and reverse 5′-CAGCTTCTGAGTTTGGTGATA-3′; mouse TNF-α, forward 5′-CCCTCACACTCAGATCATCTTCT-3 and reverse 5′-GCTACGACGTGGGCTACAG-3′; mouse 18s, forward 5′-CGGCTACCACATCCAAGGAA-3′ and reverse 5′-GCTGGAATTACCGCGGCT-3′; human VCAM-1, forward 5′-AAGGATGCGGGAGTATATGAATG-3′ and reverse 5′-GGATGCAAAATAGAGCACGAGA-3′; ICAM-1, forward 5′-GCCGGCCAGCTTATACACAA-3′ and reverse 5′-CAATCCCTCTCGTCC AGTCG-3′; human E selectin, forward 5′-TTAGGGTGCTCTGGAAGAGAGG-3′ and reverse 5′-GAGCAGCTTTGGCAATTACTGA-3′; and human 18s, forward 5′-GGCCCTGTAATTGGAATGAGTC-3′ and reverse 5′-CCAAGATCCAACTACGAGCTT-3′.

### Plasma XOR activity in morbidly obese patients who underwent bariatric surgery.

We enrolled 15 obese patients who underwent laparoscopic sleeve gastrectomy at Osaka University Hospital between April 2017 and February 2019. Of these patients, 3 were lost to follow-up during the study period. Thus, we included the remaining 12 patients in the analysis. No patient was treated with XOR inhibitors before surgery. Physical examination and metabolic parameters were measured on admission and 1 week and 1 year after the surgery. Venous blood samples were collected under fasting conditions and stored at −80°C until analysis. Plasma XOR activity was measured using the LC/TQMS method. Plasma samples from healthy volunteers were also used for the assessment of purine catabolic reactions as described above.

### Statistics.

Data are expressed as mean ± SEM, mean ± SD, or medians (IQR). Non-normally distributed variables were log-transformed before analysis. Differences between the 2 groups were analyzed by Student’s *t* test. A 1-way ANOVA followed by Tukey’s HSD test or Dunnett’s test (for comparison with the control group) was performed for multiple comparisons. The relationships between 2 continuous variables were analyzed using scatter plots and Pearson’s correlation coefficient. Changes in each clinical parameter from baseline to 1 week or 1 year after bariatric surgery were subjected to the 2-tailed paired *t* test. *P* values of less than 0.05 were considered significant. All analyses were performed using JMP Software 13.0 (SAS Institute).

### Study approval.

This study was approved by the Human Ethics Committee of Osaka University (14064-6 and 16374-6) and was conducted following the Declaration of Helsinki. Written informed consent was received from all of the study subjects. The animal experimental protocol was approved by the ethics review committee for animal experimentation of Osaka University School of Medicine. This study also conformed to the *Guide for the Care and Use of Laboratory Animals* published by the NIH (National Academies Press, 2011).

## Author contributions

YK and YF acquired and analyzed the data and wrote the manuscript. YF and HN conceived the study. YK, YF, and HN wrote the manuscript. TN, SA, and TM measured XOR activity and purine metabolites by the LC/TQMS method. TS and YM performed bariatric surgery. HN, SF, SK, and NK assessed the data. YD, NM, and IS reviewed the manuscript. All authors read and approved the final manuscript.

## Supplementary Material

Supplemental data

## Version 1 09/08/2021

Electronic publication

## References

[1] Kamei K (2014). A slight increase within the normal range of serum uric acid and the decline in renal function: associations in a community-based population. Nephrol Dial Transplant.

[2] Zhu P (2014). Serum uric acid is associated with incident chronic kidney disease in middle-aged populations: a meta-analysis of 15 cohort studies. PLoS One.

[3] Zhao G (2013). Baseline serum uric acid level as a predictor of cardiovascular disease related mortality and all-cause mortality: a meta-analysis of prospective studies. Atherosclerosis.

[4] Zhang W (2016). Serum uric acid and mortality form cardiovascular disease: EPOCH-JAPAN Study. J Atheroscler Thromb.

[5] Hisatome I (2020). Japanese society of gout and uric & nucleic acids 2019 guidelines for management of hyperuricemia and gout 3rd edition. Gout and Uric & Nucleic Acids.

[6] Xu L (2017). Recent advances on uric acid transporters. Oncotarget.

[7] Matsuura F (1998). Effect of visceral fat accumulation on uric acid metabolism in male obese subjects: visceral fat obesity is linked more closely to overproduction of uric acid than subcutaneous fat obesity. Metabolism.

[8] Liu P (2015). The effects of allopurinol on the carotid intima-media thickness in patients with type 2 diabetes and asymptomatic hyperuricemia: a three-year randomized parallel-controlled study. Intern Med.

[9] Higgins P (2014). Allopurinol reduces brachial and central blood pressure, and carotid intima-media thickness progression after ischaemic stroke and transient ischaemic attack: a randomised controlled trial. Heart.

[10] Tanaka A (2020). Febuxostat does not delay progression of carotid atherosclerosis in patients with asymptomatic hyperuricemia: a randomized, controlled trial. PLoS Med.

[11] Nagao H (2018). Hypoxanthine secretion from human adipose tissue and its increase in hypoxia. Obesity (Silver Spring).

[12] Engerson TD (1987). Conversion of xanthine dehydrogenase to oxidase in ischemic rat tissues. J Clin Invest.

[13] Brass CA (1991). Enhanced activity of the free radical producing enzyme xanthine oxidase in hypoxic rat liver. Regulation and pathophysiologic significance. J Clin Invest.

[14] Battelli MG (1992). Effects of hypoxia and ethanol on xanthine oxidase of isolated rat hepatocytes: conversion from D to O form and leakage from cells. Chem Biol Interact.

[15] Kooij A (1994). Conversion of xanthine dehydrogenase into xanthine oxidase in rat liver and plasma at the onset of reperfusion after ischemia. Hepatology.

[16] Houston M (1999). Binding of xanthine oxidase to vascular endothelium. Kinetic characterization and oxidative impairment of nitric oxide-dependent signaling. J Biol Chem.

[17] Adachi T (1993). Binding of human xanthine oxidase to sulphated glycosaminoglycans on the endothelial-cell surface. Biochem J.

[18] Meneshian A, Bulkley GB (2002). The physiology of endothelial xanthine oxidase: from urate catabolism to reperfusion injury to inflammatory signal transduction. Microcirculation.

[19] Battelli MG (2014). Pathophysiology of circulating xanthine oxidoreductase: new emerging roles for a multi-tasking enzyme. Biochim Biophys Acta.

[20] Kelley EE (2015). A new paradigm for XOR-catalyzed reactive species generation in the endothelium. Pharmacol Rep.

[21] Murase T (2016). A highly sensitive assay of human plasma xanthine oxidoreductase activity using stable isotope-labeled xanthine and LC/TQMS. J Chromatogr B Analyt Technol Biomed Life Sci.

[22] Washio KW (2017). Xanthine oxidoreductase activity is correlated with insulin resistance and subclinical inflammation in young humans. Metabolism.

[23] Furuhashi M (2018). Plasma xanthine oxidoreductase activity as a novel biomarker of metabolic disorders in a general population. Circ J.

[24] Kawachi Y (2021). Plasma xanthine oxidoreductase activity in Japanese patients with type 2 diabetes across hospitalized treatment. J Diabetes Investig.

[25] Tsushima Y (2013). Uric acid secretion from adipose tissue and its increase in obesity. J Biol Chem.

[26] Matsumoto M (2013). An improved mouse model that rapidly develops fibrosis in non-alcoholic steatohepatitis. Int J Exp Pathol.

[27] Oni ET (2013). A systematic review: burden and severity of subclinical cardiovascular disease among those with nonalcoholic fatty liver; should we care?. Atherosclerosis.

[28] Ampuero J (2015). Association of NAFLD with subclinical atherosclerosis and coronary-artery disease: meta-analysis. Rev Esp Enferm Dig.

[29] Wu S (2016). Association of non-alcoholic fatty liver disease with major adverse cardiovascular events: a systematic review and meta-analysis. Sci Rep.

[30] Gomez D, Owens GK (2012). Smooth muscle cell phenotypic switching in atherosclerosis. Cardiovasc Res.

[31] Shankman LS (2015). KLF4-dependent phenotypic modulation of smooth muscle cells has a key role in atherosclerotic plaque pathogenesis. Nat Med.

[32] Nakamura T (2019). The influence of albumin on the plasma xanthine oxidoreductase inhibitory activity of allopurinol, febuxostat and topiroxostat: insight into extra-urate lowering effect. Integr Mol Med.

[33] Furuhashi M (2019). Annual change in plasma xanthine oxidoreductase activity is associated with changes in liver enzymes and body weight. Endocr J.

[34] Xu C (2015). Xanthine oxidase in non-alcoholic fatty liver disease and hyperuricemia: one stone hits two birds. J Hepatol.

[35] Nakatsu Y (2015). The xanthine oxidase inhibitor febuxostat suppresses development of nonalcoholic steatohepatitis in a rodent model. Am J Physiol Gastrointest Liver Physiol.

[36] Byrne CD, Targher G (2015). NAFLD: a multisystem disease. J Hepatol.

[37] Adams LA (2017). Non-alcoholic fatty liver disease and its relationship with cardiovascular disease and other extrahepatic diseases. Gut.

[38] Lim S (2019). Crosstalk between nonalcoholic fatty liver disease and cardiometabolic syndrome. Obes Rev.

[39] Nakamura T (2016). Effects of topiroxostat and febuxostat on urinary albumin excretion and plasma xanthine oxidoreductase activity in db/db mice. Eur J Pharmacol.

[40] Kario K (2021). Comparative effects of topiroxostat and febuxostat on arterial properties in hypertensive patients with hyperuricemia. J Clin Hypertens (Greenwich).

[41] Wirka RC (2019). Atheroprotective roles of smooth muscle cell phenotypic modulation and the TCF21 disease gene as revealed by single-cell analysis. Nat Med.

[42] Li PF (1997). Differential effect of hydrogen peroxide and superoxide anion on apoptosis and proliferation of vascular smooth muscle cells. Circulation.

[43] Furuhashi M (2020). Differential regulation of hypoxanthine and xanthine by obesity in a general population. J Diabetes Investig.

[44] Kushiyama A (2012). Xanthine oxidoreductase is involved in macrophage foam cell formation and atherosclerosis development. Arterioscler Thromb Vasc Biol.

[45] Nomura J (2014). Xanthine oxidase inhibition by febuxostat attenuates experimental atherosclerosis in mice. Sci Rep.

[46] Ives A (2015). Xanthine oxidoreductase regulates macrophage IL1β secretion upon NLRP3 inflammasome activation. Nat Commun.

[47] Nakagami F (2010). Estrogen attenuates vascular remodeling in Lp(a) transgenic mice. Atherosclerosis.

[48] Fujishima Y (2017). Adiponectin association with T-cadherin protects against neointima proliferation and atherosclerosis. FASEB J.

[49] Mori T (2013). A novel role for adipose ephrin-B1 in inflammatory response. PLoS One.

