# Supplementary material for: Increased plasma XOR activity induced by NAFLD/NASH and its possible involvement in vascular neointimal proliferation
Source: JCI Insight. 2021 Sep 8;6(17):e144762. doi: 10.1172/jci.insight.144762 (PMC8492303; doi:10.1172/jci.insight.144762)
Supplement: Supplemental data [file jciinsight-6-144762-s080.pdf]

Supplemental Table 1. Baseline clinical characteristics of morbidly obese patients who underwent bariatric surgery.

**Clinical parameters**	
**n (males/females)**	**12 (8/4)**
**Age (years)**	**46.9 ± 5.9**
**BW (kg)**	**111.5 ± 14.1**
**BMI (kg/m^2)**	**38.3 ± 4.5**
**FPG (mg/dL)**	**96.6 ± 14.3**
**HbA1c (%)**	**6.0 ± 1.0**
**T-cho (mg/dL)**	**196.3 ± 33.7**
**LDL-cho (mg/dL)**	**130.7 ± 38.2**
**HDL-cho (mg/dL)**	**48.1 ± 12.1**
**TG (mg/dL)**	**132.5 (91.5-156.3)**
**AST (IU/L)**	**23.0 (18.3-27.8)**
**ALT (IU/L)**	**33.5 (17.5-38.8)**
**γ-GTP (IU/L)**	**40.5 (22.5-53.0)**
**Creatinine (mg/dL)**	**0.83 ± 0.21**
**eGFR (ml/min/1.73m^2)**	**74.1 ± 13.2**
**UA (mg/dL)**	**6.9 ± 1.8**
**hs-CRP (mg/dL)**	**0.18 (0.12-0.34)**
**Adiponectin (μg/mL)**	**6.3 (4.3-9.4)**
**XOR activity (pmol/h/mL)**	**129.0 (56.7-375.5)**
**Complication of type 2 diabetes**	**50 (%)**
**Complication of hypertension**	**50 (%)**
**Complication of dyslipidemia**	**67 (%)**

Data are presented as means ± SD, medians (IQRs), or the number of subjects (%). BW, body weight; BMI, body mass index; FPG, fasting plasma glucose; T-cho, total-cholesterol; LDL-cho, low-density lipoprotein-cholesterol; HDL-cho, high-density lipoprotein-cholesterol; TG, triglyceride; AST, aspartate transaminase; ALT, alanine transaminase; γ-GTP, γ-glutamyl transpeptidase; eGFR, estimated glomerular filtration rate; UA, uric acid; hs-CRP, high sensitivity C reactive protein; XOR, xanthine oxidoreductase.

Supplemental Table 2. Correlations between baseline plasma XOR activity and other clinical parameters.

Clinical parameters	R	*p* value
Sex	0.31	0.322
Age	-0.12	0.709
BW	-0.51	0.087
BMI	-0.22	0.485
FPG	0.16	0.619
HbA1c	0.18	0.573
T-cho	0.20	0.531
LDL-cho	0.11	0.725
HDL-cho	0.09	0.779
Log-TG	-0.14	0.671
Log-AST	0.92	<0.0001
Log-ALT	0.95	<0.0001
Log-γ-GTP	0.26	0.414
Creatinine	0.002	0.995
eGFR	-0.15	0.639
UA	0.18	0.574
Log-hs-CRP	-0.56	0.060
Log-Adiponectin	-0.29	0.353
Complication of type 2 diabetes	0.15	0.638
Complication of hypertension	0.20	0.537
Complication of dyslipidemia	0.29	0.366

BW, body weight; BMI, body mass index; FPG, fasting plasma glucose; T-cho, total-cholesterol; LDL-cho, low-density lipoprotein-cholesterol; HDL-cho, high-density lipoprotein-cholesterol; TG, triglyceride; AST, aspartate transaminase; ALT, alanine transaminase; γ-GTP, γ-glutamyl transpeptidase; eGFR, estimated glomerular filtration rate; UA, uric acid; hs-CRP, high sensitivity C reactive protein; XOR, xanthine oxidoreductase.

Supplemental Table 3. Changes in clinical parameters from baseline to 1 week or 1 year following bariatric surgery.

Clinical parameters	Pre	1 week	*p* value (vs Pre)	1 year	*p* value (vs Pre)
BW (kg)	111.5 ± 14.1	107.1 ± 12.6	0.0003	89.4 ± 14.6	<0.0001
BMI (kg/m^2)	38.3 ± 4.5	36.9 ± 4.4	<0.0001	30.7 ± 4.7	<0.0001
FPG (mg/dL)	96.6 ± 14.3	78.9 ± 17.4	0.003	95.0 ± 13.9	0.670
HbA1c (%)	6.0 ± 1.0	-	-	5.7 ± 0.9	0.046
T-cho (mg/dL)	196.3 ± 33.7	186.8 ± 34.2	0.306	198.7 ± 34.8	0.842
LDL-cho (mg/dL)	130.7 ± 38.2	128.7 ± 32.2	0.827	118.5 ± 28.8	0.244
HDL-cho (mg/dL)	48.1 ± 12.1	35.9 ± 6.9	0.001	66.8 ± 16.1	<0.0001
Log-TG (mg/dL)	4.74 ± 0.38	4.67 ± 0.24	0.505	4.52 ± 0.47	0.091
Log-AST (IU/L)	3.20 ± 0.42	3.64 ± 0.55	0.031	2.84 ± 0.17	0.017
Log-ALT (IU/L)	3.44 ± 0.68	3.84 ± 0.79	0.131	2.66 ± 0.43	0.005
Log-γ-GTP (IU/L)	3.57 ± 0.52	3.79 ± 0.72	0.145	2.97 ± 0.44	0.0003
Creatinine (mg/dL)	0.84 ± 0.21	0.84 ± 0.20	0.963	0.78 ± 1.44	0.077
eGFR (ml/min/1.73m^2)	74.1 ± 13.2	73.4 ± 11.7	0.826	77.5 ± 8.6	0.217
UA (mg/dL)	6.9 ± 1.8	8.0 ± 2.6	0.177	6.4 ± 1.6	0.393
Log-hs-CRP (mg/dL)	-1.61 ± 0.91	0.44 ± 0.62	<0.0001	-2.51 ± 1.22	0.031
Log-Adiponectin (µg/mL)	1.82 ± 0.51	1.76 ± 0.54	0.372	2.20 ± 0.56	0.021
Log-XOR (pmol/h/mL)	4.9 ± 1.3	5.3 ± 1.0	0.258	3.1 ± 0.9	0.0001

Data are presented as means ± SD. BW, body weight; BMI, body mass index; FPG, fasting plasma glucose; T-cho, total-cholesterol; LDL-cho, low-density lipoprotein-cholesterol; HDL-cho, high-density lipoprotein-cholesterol; TG, triglyceride; AST, aspartate transaminase; ALT, alanine transaminase; γ-GTP, γ-glutamyl transpeptidase; eGFR, estimated glomerular filtration rate; UA, uric acid; hs-CRP, high sensitivity C reactive protein; XOR, xanthine oxidoreductase.

Supplemental Table 4. Correlations between changes in plasma XOR activity and those in other clinical parameters at 1 week and 1 year following bariatric surgery.

	1 week		1 year	
**Clinical parameters**	**R**	***p* value**	**R**	***p* value**
**ΔBW**	**-0.52**	**0.084**	**0.27**	**0.390**
**ΔBMI**	**-0.53**	**0.079**	**0.27**	**0.405**
**ΔFPG**	**-0.35**	**0.268**	**0.61**	**0.034**
**ΔHbA1c**	**-**	**-**	**0.54**	**0.068**
**ΔT-cho**	**-0.45**	**0.017**	**0.22**	**0.484**
**ΔLDL-cho**	**-0.16**	**0.614**	**0.34**	**0.287**
**ΔHDL-cho**	**0.10**	**0.754**	**-0.25**	**0.434**
**ΔLog-TG**	**-0.36**	**0.252**	**0.33**	**0.293**
**ΔLog-AST**	**0.94**	**<0.0001**	**0.76**	**0.004**
**ΔLog-ALT**	**0.91**	**<0.0001**	**0.80**	**0.002**
**ΔLog-γ-GTP**	**0.49**	**0.108**	**0.48**	**0.117**
**ΔCreatinine**	**0.19**	**0.549**	**0.27**	**0.398**
**ΔeGFR**	**-0.24**	**0.455**	**-0.25**	**0.440**
**ΔUA**	**0.15**	**0.643**	**0.22**	**0.498**
**ΔLog-hs-CRP**	**-0.30**	**0.336**	**-0.22**	**0.499**
**ΔLog-Adiponectin**	**0.25**	**0.435**	**-0.41**	**0.182**

BW, body weight; BMI, body mass index; FPG, fasting plasma glucose; T-cho, total-cholesterol; LDL-cho, low-density lipoprotein-cholesterol; HDL-cho, high-density lipoprotein-cholesterol; TG, triglyceride; AST, aspartate transaminase; ALT, alanine transaminase; γ-GTP, γ-glutamyl transpeptidase; eGFR, estimated glomerular filtration rate; UA, uric acid; hs-CRP, high sensitivity C reactive protein; XOR, xanthine oxidoreductase.

Supplementary Figure Legends

Supplemental Figure 1. Increased liver XOR in NAFLD/NASH mice fed CDAHFD.

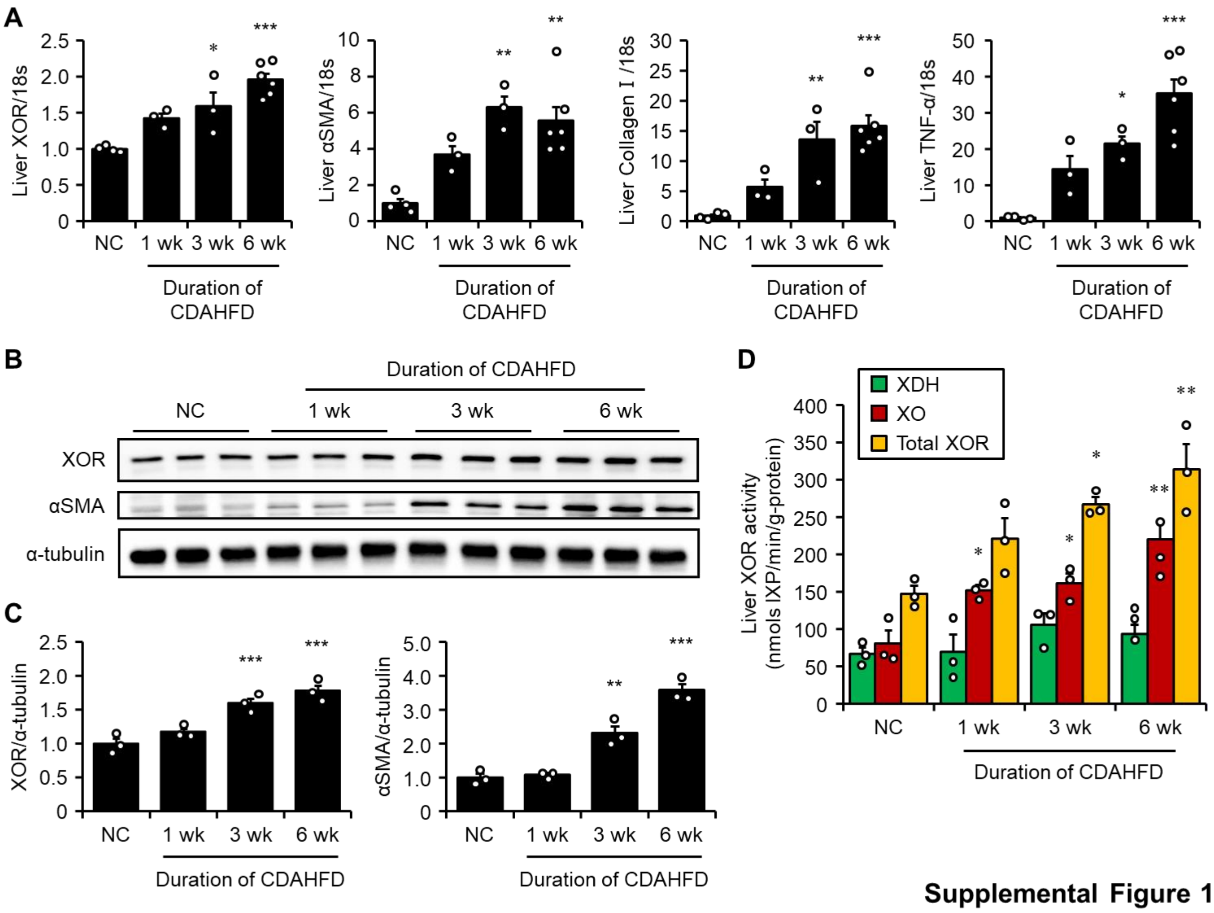

Supplemental Figure 1

Male C57BL/6J mice were fed a choline-deficient, l-amino acid-defined, high-fat diet (CDAHFD) for up to 6 weeks from 8 weeks of age. **(A)** Relative mRNA levels of XOR, αSMA, collagen type I, and TNF-α in liver. n=4; normal chow (NC) for 6 weeks, n=3; CDAHFD for 1 week and 3 weeks and, n=6; CDAHFD for 6 weeks. **(B)** Immunoblots for XOR, αSMA, and α-tubulin in liver. **(C)** Relative protein levels of XOR and αSMA normalized to α-tubulin. n=3 for each group. **(D)** Liver XDH, XO, and total XOR activity after feeding with CDAHFD. The HPLC-FLD method was used to distinguish between XO activity and total XOR (XO + XDH) activity. XDH activity was calculated by subtracting XO activity from total XOR activity. n=3 for each group. Data are the mean ± SEM. $^{*}P < 0.05$, $^{**}P < 0.01$, and $^{***}P < 0.001$ vs NC (one-way ANOVA with Dunnett's post hoc test).

Supplemental Figure 2. Body weight, liver weight, and serum lipid profiles in NASH mice treated with topiroxostat.

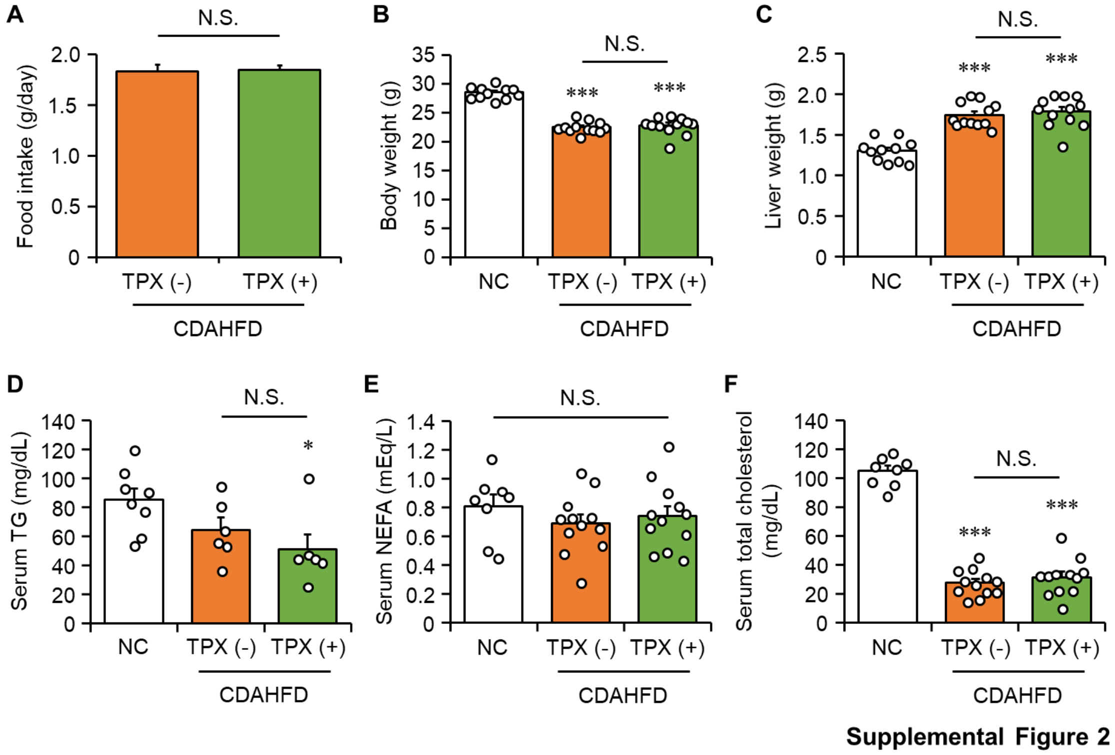

Male C57BL/6J mice were divided into three groups; fed normal chow (NC) or fed a choline-deficient, l-amino acid-defined, high-fat diet (CDAHFD) treated with or without topiroxostat (TPX) (1 mg/kg/day). Topiroxostat was administered 4 weeks after feeding with CDAHFD, and all mice were examined 8 weeks after feeding with each diet. **(A)** Food intake in mice fed CDAHFD treated with or without topiroxostat. n=11 for NC, n=12 for CDAHFD/TPX (-), and n=13 for CDAHFD/TPX (+). N.S., not significant (unpaired t test). **(B and C)** Body weight **(B)** and liver weight **(C)** in each group. n=11 for NC, n=12 for CDAHFD/TPX (-), and n=13 for CDAHFD/TPX (+). **(D-F)** Serum triglyceride (TG) **(D)**, serum non-esterified fatty acids (NEFA) **(E)**, and serum total cholesterol **(F)** levels in each group. n=8 for NC and n=6 for CDAHFD/TPX (-) and CDAHFD/TPX (+). Data are the mean ± SEM; $^{*}P < 0.05$, $^{**}P < 0.01$, and $^{***}P < 0.001$ vs NC. N.S., not significant (one-way ANOVA with Tukey's post hoc test).

Supplemental Figure 3. Effects of topiroxostat on hepatic steatosis, XOR activity, and purine metabolites in livers of NASH mice.

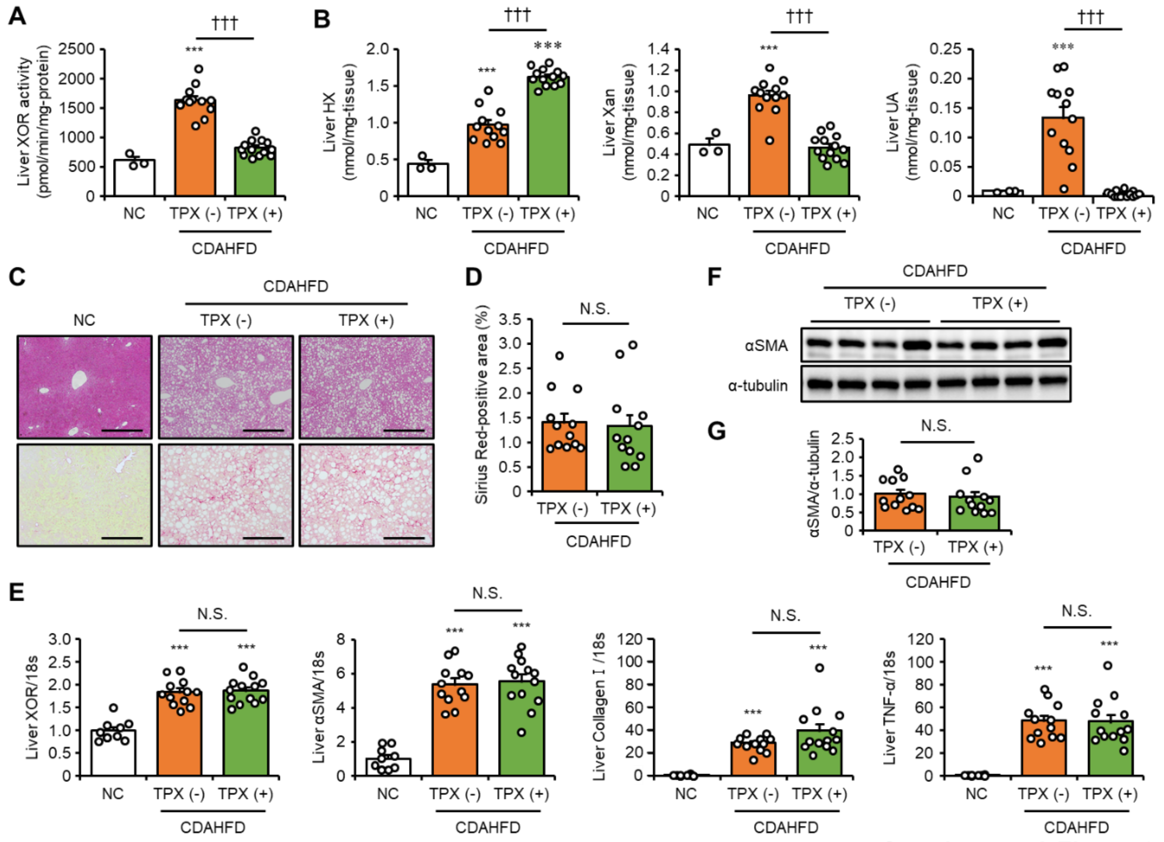

Supplemental Figure 3

Male C57BL/6J mice were divided into three groups; fed normal chow (NC) or fed a choline-deficient, l-amino acid-defined, high-fat diet (CDAHFD) treated with or without topiroxostat (TPX) (1 mg/kg/day). Topiroxostat was administered 4 weeks after feeding with CDAHFD, and all mice were examined 8 weeks after feeding with each diet. **(A)** Liver XOR activity measured by the LC/TQMS method in each group. n=11 for NC, n=12 for CDAHFD/TPX (-), and n=13 for CDAHFD/TPX (+). **(B)** Liver concentrations of hypoxanthine (HX), xanthine (Xan), and uric acid (UA) in each group. n=3 for NC, n=12 for CDAHFD/TPX (-), and n=13 for CDAHFD/TPX (+). Data are the mean ± SEM; $^{*}P < 0.05$, $^{**}P < 0.01$, and $^{***}P < 0.001$ vs NC and $^{†}P < 0.01$, $^{††}P < 0.01$, and $^{†††}P < 0.001$. N.S., not significant (one-way ANOVA with Tukey’s post hoc test). **(C)** Representative hematoxylin and eosin (H&E) (upper panels) and

Sirius Red (lower panels)-stained sections of livers obtained from each group. Scale bars = 200 μm. **(D)** The percent Sirius Red-positive area was calculated from three randomly selected sections for each mouse. n=12 for each group. N.S., not significant (unpaired t test). **(E)** Relative mRNA levels of XOR, αSMA, collagen type I, and TNF-α in livers of each group. n=10 for NC, n=12 for CDAHFD/TPX (-), and n=13 for CDAHFD/TPX (+). N.S., not significant (one-way ANOVA with Tukey's post hoc test). **(F)** Immunoblots for αSMA and α-tubulin in liver. **(G)** Relative protein levels of αSMA normalized to α-tubulin. n=12 for each group. N.S., not significant (unpaired t test).

Supplemental Figure 4. Effects of by-products of XOR, xanthine or uric acid, on proliferation of HASMCs and effects of liver S9-derived XOR on expression of adhesion molecules in HUVECs.

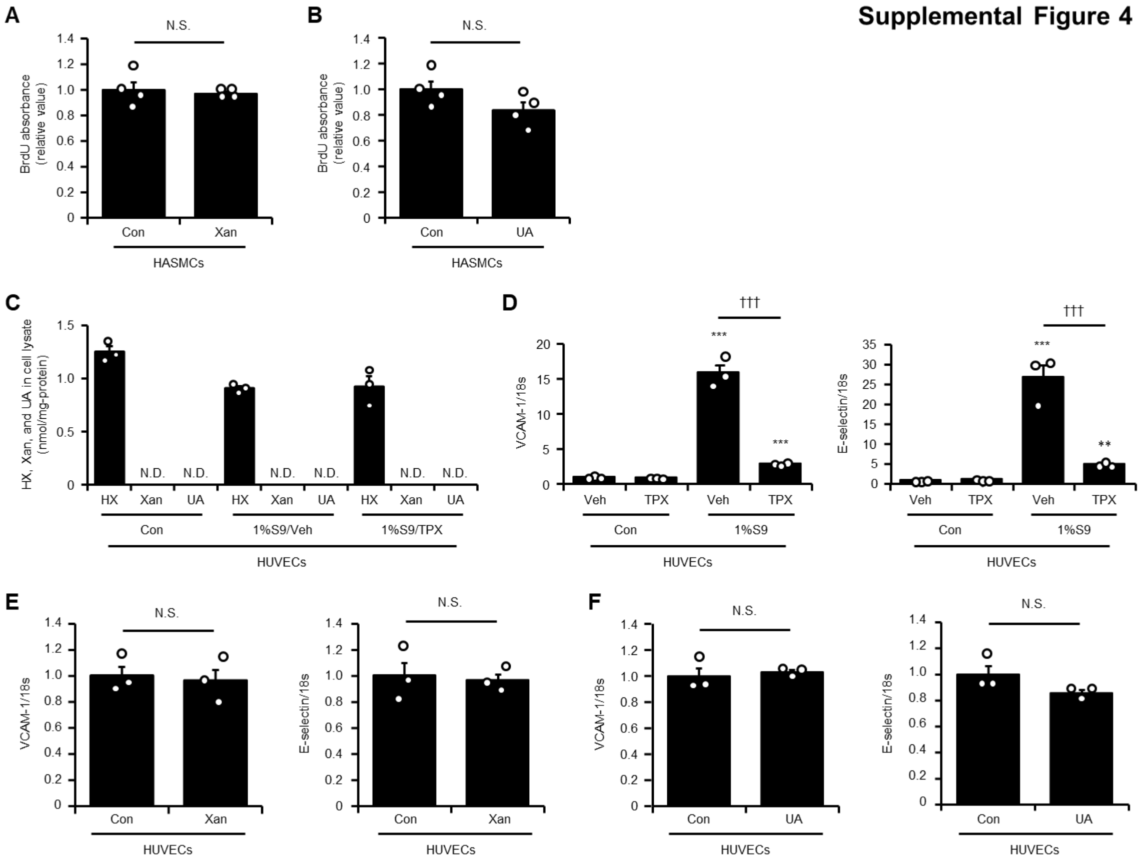

(A) and (B) After 24 hrs starvation, human aortic smooth muscle cells (HASMCs) were incubated with or without 20 μM of xanthine (Xan) **(A)** or uric acid (UA) **(B)** for 24hrs, and then labeled with BrdU for additional 18 hrs. Shown are relative incorporation of BrdU. n=4 for each group. Data are the mean ± SEM. Con, control. N.S., not significant (unpaired t test). **(C)** and **(D)** Human umbilical vein endothelial cells (HUVECs) were incubated with or without 1 % human liver S9 compartment (S9) in the presence or absence of 10 μM topiroxostat (TPX) for 4 hrs. **(C)** Concentrations of hypoxanthine (HX), Xan, and UA in cell lysates. n=3 for each group. **(D)** Relative mRNA levels of VCAM-1 and E-selectin. n=3 for each group. Data are the mean ± SEM; $^{*}P < 0.05$, $^{**}P < 0.01$, and $^{***}P < 0.001$ vs control (Con, without 1% S9) and $^{\dagger}P < 0.01$, $^{\dagger\dagger}P < 0.01$, and $^{\dagger\dagger\dagger}P < 0.001$ (one-way ANOVA with Tukey's post hoc test). N.D., not

detected. **(E)** and **(F)** HUVECs were incubated with or without 20 μM of Xan **(E)** or UA **(F)** for 4 hrs. Shown are relative mRNA levels of VCAM-1 and E-selectin. n=3 for each group. Data are the mean ± SEM. Con, control. N.S., not significant (unpaired t test).

Supplemental Figure 5. The graphical abstract of this article.

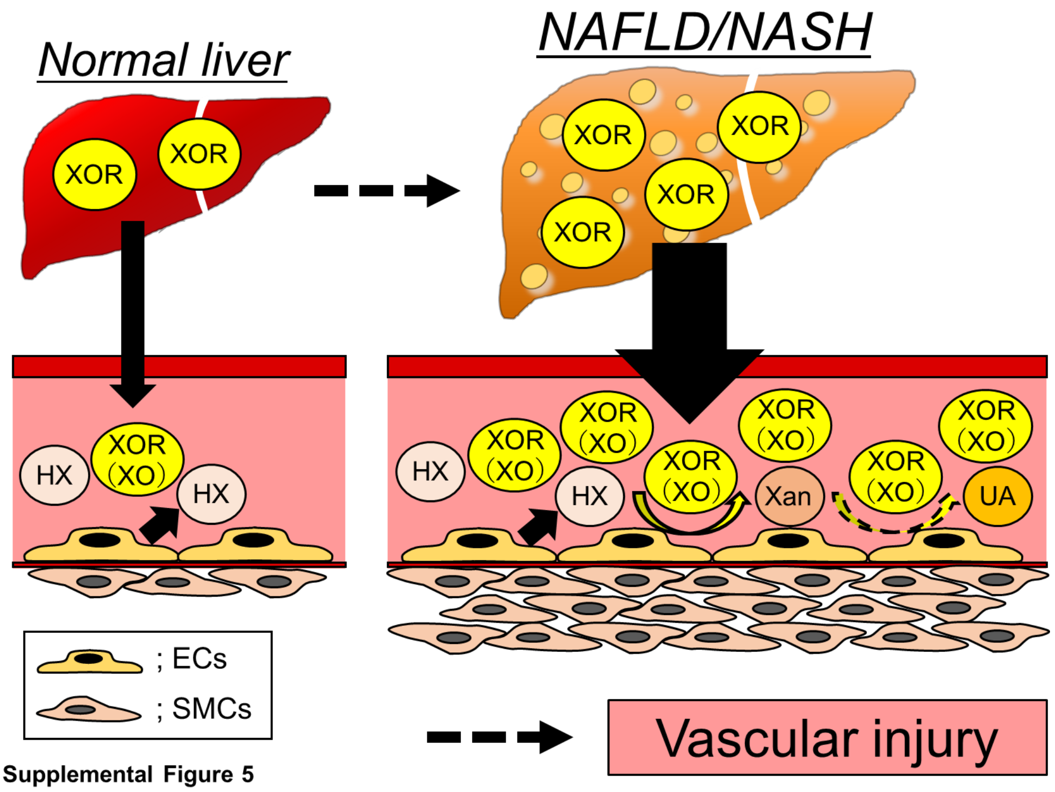

Supplemental Figure 5

In NAFLD/NASH, plasma XOR (mainly XO) activity is increased due to excess leakage of hepatic XOR from the damaged liver into the circulation, together with its upregulation in liver. Hypoxanthine (HX), a substrate for XOR, is potentially derived from vascular endothelial cells (ECs) and other cells, including adipocytes, and subsequently catabolized to at least xanthine (Xan) by markedly high plasma XOR activity. Such local purine catabolic reactions in the bloodstream may be involved in the initial cascade of vascular injury and subsequent proliferation of vascular smooth muscle cells (SMCs) in NAFLD/NASH conditions.
